# Early exposure to broadly neutralizing antibodies may trigger a dynamical switch from progressive disease to lasting control of SHIV infection

**DOI:** 10.1371/journal.pcbi.1008064

**Published:** 2020-08-20

**Authors:** Rajat Desikan, Rubesh Raja, Narendra M. Dixit

**Affiliations:** 1 Department of Chemical Engineering, Indian Institute of Science, Bengaluru, India; 2 Centre for Biosystems Science and Engineering, Indian Institute of Science, Bengaluru, India; Columbia University Medical Center, UNITED STATES

## Abstract

Antiretroviral therapy (ART) for HIV-1 infection is life-long. Stopping therapy typically leads to the reignition of infection and progressive disease. In a major breakthrough, recent studies have shown that early initiation of ART can lead to sustained post-treatment control of viremia, raising hopes of long-term HIV-1 remission. ART, however, elicits post-treatment control in a small fraction of individuals treated. Strikingly, passive immunization with broadly neutralizing antibodies (bNAbs) of HIV-1 early in infection was found recently to elicit long-term control in a majority of SHIV-infected macaques, suggesting that HIV-1 remission may be more widely achievable. The mechanisms underlying the control elicited by bNAb therapy, however, remain unclear. Untreated infection typically leads to progressive disease. We hypothesized that viremic control represents an alternative but rarely realized outcome of the infection and that early bNAb therapy triggers a dynamical switch to this outcome. To test this hypothesis, we constructed a model of viral dynamics with bNAb therapy and applied it to analyse clinical data. The model fit quantitatively the complex longitudinal viral load data from macaques that achieved lasting control. The model predicted, consistently with our hypothesis, that the underlying system exhibited bistability, indicating two potential outcomes of infection. The first had high viremia, weak cytotoxic effector responses, and high effector exhaustion, marking progressive disease. The second had low viremia, strong effector responses, and low effector exhaustion, indicating lasting viremic control. Further, model predictions suggest that early bNAb therapy elicited lasting control via pleiotropic effects. bNAb therapy lowers viremia, which would also limit immune exhaustion. Simultaneously, it can improve effector stimulation via cross-presentation. Consequently, viremia may resurge post-therapy, but would encounter a primed effector population and eventually get controlled. ART suppresses viremia but does not enhance effector stimulation, explaining its limited ability to elicit post-treatment control relative to bNAb therapy.

## Author summary

In a remarkable advance in HIV cure research, a recent study showed that 3 weekly doses of HIV-1 broadly neutralizing antibodies (bNAbs) soon after infection kept viral levels controlled for years in most macaques treated. If translated to humans, this bNAb therapy may elicit a functional cure, or long-term remission, of HIV-1 infection, eliminating the need for life-long antiretroviral therapy (ART). How early bNAb therapy works remains unknown. Here, we elucidate the mechanism using mathematical modeling and analysis of *in vivo* data. We predict that early bNAb therapy suppresses viremia, which reduces exhaustion of cytotoxic effector cells, and enhances antigen uptake and effector stimulation. Collectively, these effects drive infection to lasting control. Model predictions based on these effects fit *in vivo* data quantitatively. ART controls viremia but does not improve effector stimulation, explaining its weaker ability to induce lasting control post-treatment. Our findings may help improve strategies for achieving functional cure of HIV-1 infection.

## Introduction

Current antiretroviral therapies (ART) for HIV-1 infection control viremia in infected individuals but are unable to eradicate the virus [1]. A reservoir of latently infected cells, which is established soon after infection [2], escapes drugs and the host immune response [3], is long-lived [4, 5], and can reignite infection following the cessation of therapy [6], presents the key obstacle to sterilizing cure. Efforts are now aimed at eliciting a “functional cure” of the infection, where the virus can be controlled without life-long treatment even though eradication is not possible [7]. That functional cure can be achieved has been demonstrated by the VISCONTI trial, where a subset of patients, following early initiation of ART, maintained undetectable viremia long after the cessation of treatment [8]. A limitation, however, is that the subset that achieves post-treatment control with ART is small, 5-15% of the patients treated [9]. In a major advance, Nishimura *et al*. [10] found recently that early, short-term passive immunization with a combination of two HIV-1 broadly neutralizing antibodies (bNAbs) elicited lasting control of viremia in 10 of 13, or nearly 77%, of SHIV-infected macaques treated. This high success rate raises the prospect of achieving functional cure in all HIV-1 infected individuals using short-term bNAb therapy. Efforts have been initiated to develop immunotherapies that may further improve response rates in primate models [11–14] and to translate them to humans [15–17].

Devising passive immunization protocols that would maximize the chances of achieving functional cure requires an understanding of the mechanism(s) with which early, short-term passive immunization with bNAbs induces sustained control of viremia. Nishimura *et al*. [10] argue that the control they observed, lasting long (years) after the administered bNAbs were cleared from circulation (weeks), was due to the effector function of cytotoxic cells such as CD8^+^ T lymphocytes (CTLs) because transient suppression of effector cells, using anti-CD8 antibodies (Abs), well after the establishment of control resulted in a temporary surge of viremia. How short-term bNAb therapy leads to sustained effector stimulation, and therefore lasting viremic control, remains unknown [15].

Here, we propose a dynamical systems-based role of bNAbs in eliciting lasting control. We hypothesized that lasting viremic control is a distinct but rarely realized outcome of HIV-1 infection and that early, short-term bNAb therapy triggers a switch in disease dynamics that significantly increases the likelihood of realizing this outcome. To test this hypothesis and to elucidate the mechanisms with which bNAbs orchestrate this switch, we constructed a mathematical model of HIV dynamics under bNAb therapy and applied it to analyse recent *in vivo* data.

## Results

### Mathematical model of viral dynamics with passive immunization

**Fig 1:**
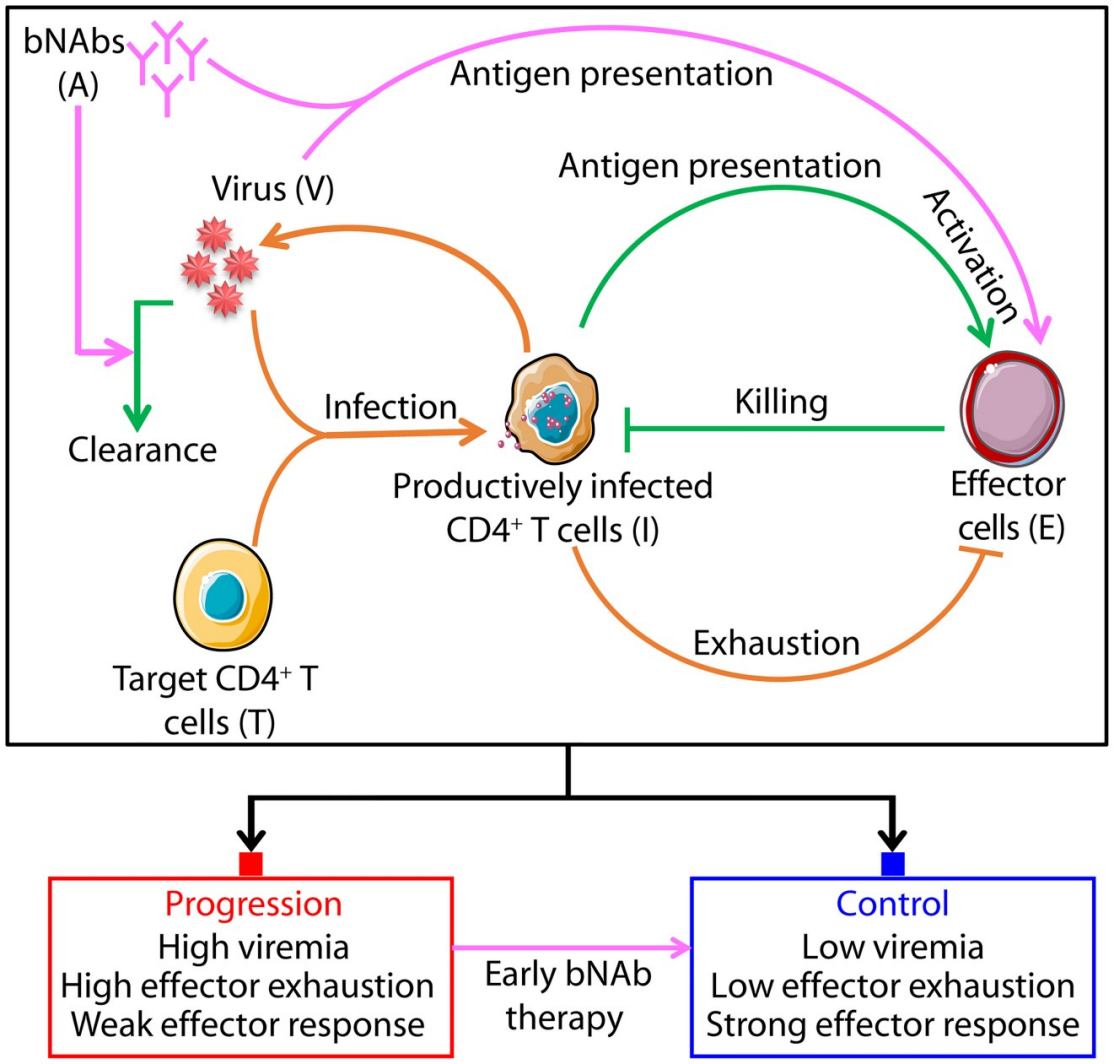
Schematic of the mathematical model of SHIV dynamics with bNAb therapy. Orange and green arrows indicate processes involved in the growth and control of infection, respectively, while magenta arrows indicate processes initiated or enhanced by bNAbs. Corresponding model equations are described in Methods. Steady state analysis of the model indicates two outcomes of the infection: chronic infection with high viremia that marks progressive disease (red filled square), typically realized in the absence of treatment, and viremic control (blue filled square), a switch to which is orchestrated by early bNAb therapy.

We constructed a mathematical model that considered the within-host dynamics of populations of uninfected target CD4^+^ T cells, productively infected cells, free virions, effector cells, and administered bNAbs (Fig 1). The dynamics in the absence of bNAbs were similar to those in recent models of viral dynamics that include effector cells [18–20]. Briefly, uninfected target cells get infected by free virions to yield productively infected cells, which in turn produce more free virions. Effector cells are stimulated by infected cells and could become exhausted by sustained antigenic stimulation. Activated effectors kill infected cells. We modified these processes based on the known effects of bNAbs [15, 21]: bNAbs opsonize free virions, preventing them from infecting cells and enhancing their clearance [22–24]. Opsonized virus can also be taken up by immune cells, particularly macrophages, and via processes now being unraveled improve antigen presentation and increase infected cell death [25–28]. Thus, we let bNAbs 1) enhance the clearance of free virions and 2) increase effector activation, via enhanced antigen uptake, both in a dose-dependent manner. The enhanced viral clearance, which results in a reduction in viremia, was assumed to subsume the effect of virus neutralization (Methods). By lowering antigen levels, bNAbs could reverse effector exhaustion.

To describe the effects of ART, we let reverse transcriptase inhibitors reduce the productive infection of target cells and protease inhibitors render a fraction of progeny virions non-infectious (Methods). Anti-CD8 Abs were assumed to reduce the effector population and compromise host effector functions for a duration corresponding to the residence time of the depleting Abs in circulation.

We constructed equations to describe the resulting dynamics and solved them using parameter values representative of SHIV infection of macaques (Methods). The considerations that led us to the building up of the necessary complexity beginning with the basic model of viral dynamics and culminating in the present model are discussed in Methods.

### Model predictions fit *in vivo* data

**Fig 2:**
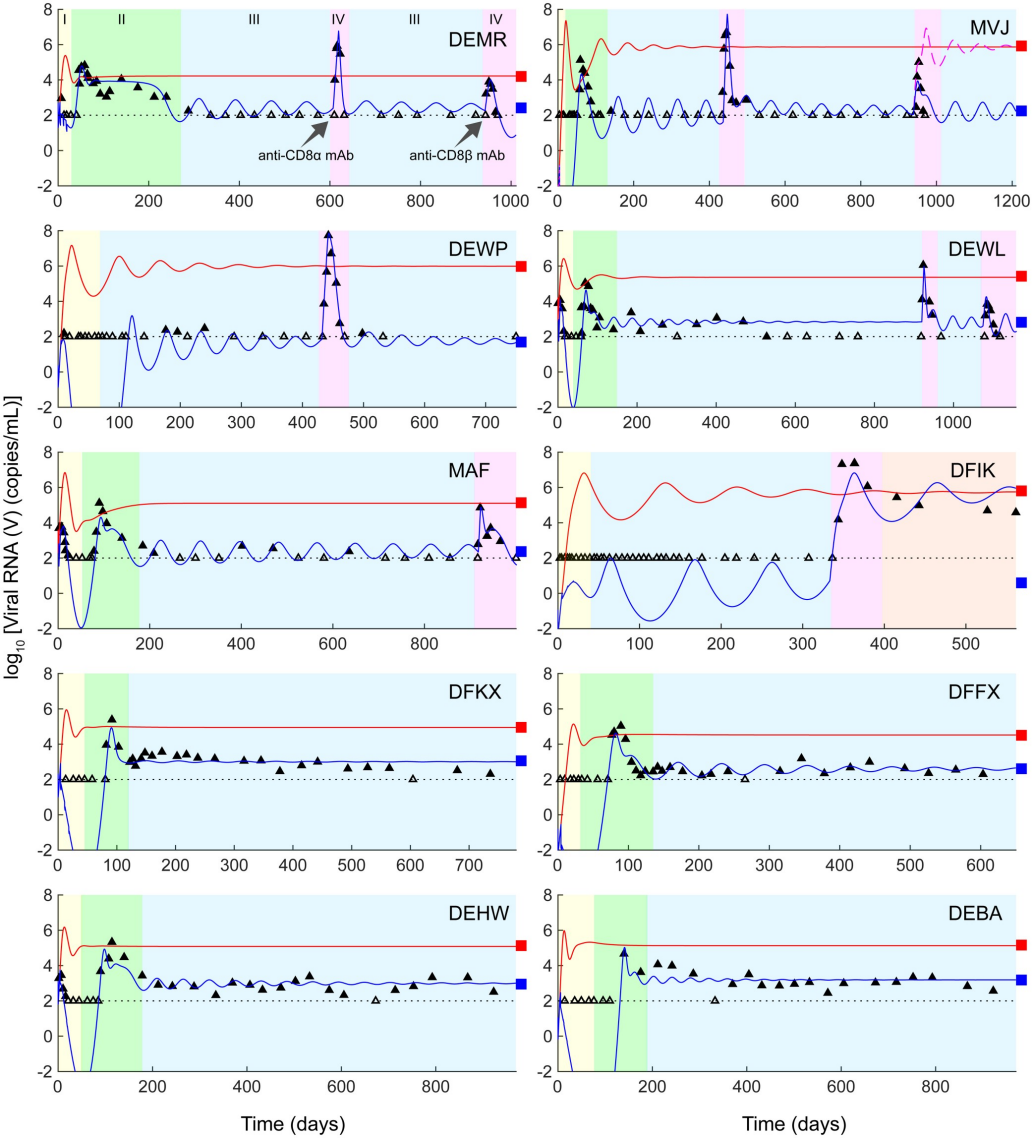
Model fits viral dynamics in responder macaques. Model fits (blue lines) to viral load data [10] (symbols) from the 10 controller macaques, shown in individual panels. Empty symbols mark measurements showing undetectable viremia and filled symbols above detection. The latter were used for fitting and the former censored. Black dotted lines in all panels indicate the viral load detection limit (100 RNA copies/mL). The corresponding bNAb concentration dynamics are in Fig 3. In all panels, phase I (yellow) marks the duration when bNAbs are present in circulation, phase II (green) the viremic resurgence post the clearance of bNAbs, phase III (blue) the ensuing viremic control, and, where relevant, phase IV (pink), in two parts, the disruption of this control using anti-CD8*α* and anti-CD8*β* Abs, respectively. For the macaque MVJ, we demonstrate the loss of viral control that occurs when effector depletion levels are increased (magenta dashed line), as is observed with the macaque DFIK. The digitized data used for the fitting is available as a supplementary excel file (S1 Data). In all cases, predictions without bNAb therapy are included for comparison (red lines). Parameter values used are in Table 1 and S1 Table. Macaques DEWP, MVJ, DFFX, DFKX, and DFIK were inoculated intrarectally and DEWL and MAF intravenously with 1000 TCID50 (50% tissue culture infective dose) of SHIVAD8-EO virus. DEMR, DEBA, and DEHW received 100 TCID_50_ intravenously. The treatments are summarized in S10 Table.

**Fig 3:**
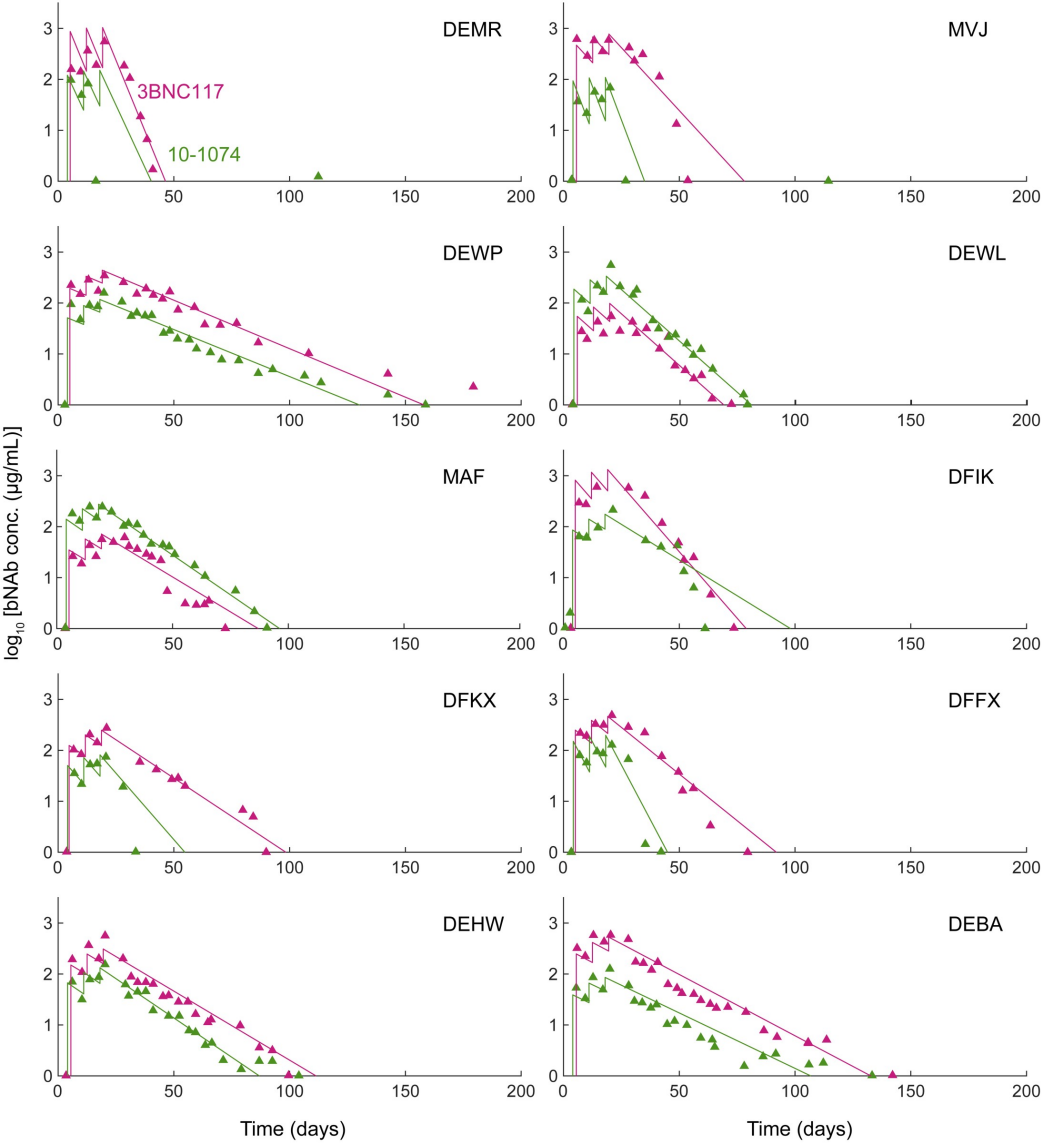
bNAb pharmacokinetics in responders. Fits of model predictions (lines) to data from individual macaques (symbols) of bNAb plasma concentrations for the ten responders in Nishimura *et al*. [10] obtained by simultaneously fitting our model (Eqs 13–20) to *V*, *A*_1_ and *A*_2_ across all macaques. Methods for the fitting procedure. The resulting parameter estimates are in S1 Table. The bNAb serum half-lives averaged across all macaques are 9.0 days (range 2.7–15.8 days) for 3BNC117, and 8.2 days (range 2.5–16.3 days) for 10-1074.

**Fig 4:**
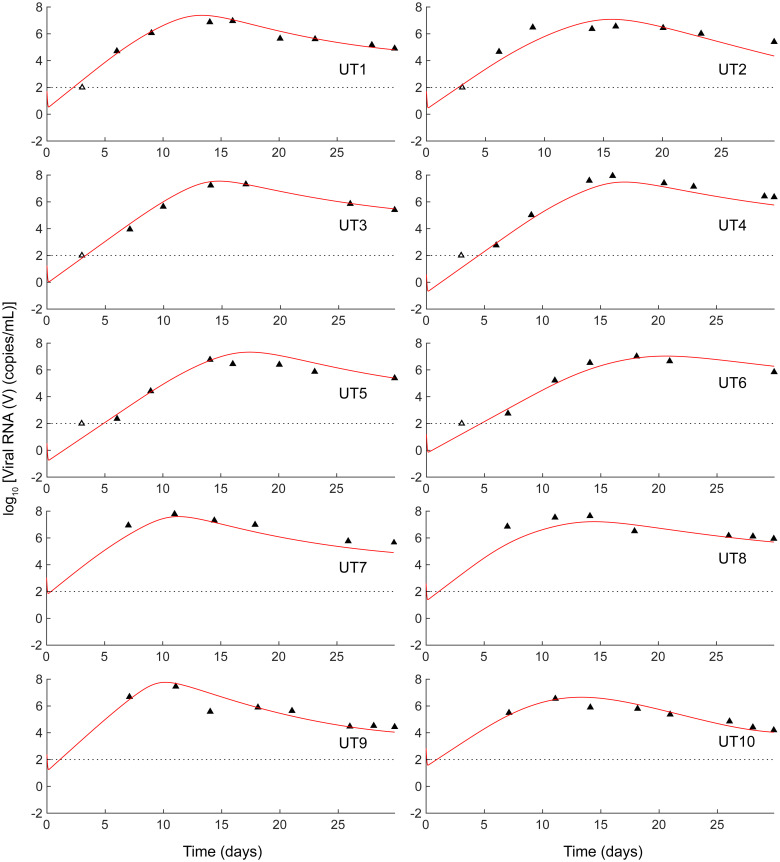
Untreated macaques. Fits (red lines) to the acute phase viral loads of the ten untreated macaques in Nishimura *et al*. [10] obtained by simultaneously fitting our model (Eqs 13–20) to *V*, *A*_1_ and *A*_2_ across all macaques. Methods for the fitting procedure. The best-fit parameter estimates are in S2 Table.

To test whether the model accurately captured the underlying dynamics of infection and the influence of bNAbs, we applied it to describe the viral load changes reported in Nishimura *et al*. [10] where 3 weekly infusions of a combination of two HIV-1 bNAbs, 3BNC117 and 10-1074, starting from day 3 post SHIV challenge were administered. We considered the 10 “responder” macaques who showed no significant decline in CD4^+^ T cell counts and included 6 with undetectable post-treatment set-point viremia (< 10^2^ copies/mL), termed “controllers”, and 4 with detectable but low post-treatment set-point viremia (< 10^3.5^ copies/mL). We also considered 10 untreated macaques in the study with viral load measurements reported during the acute phase of infection. Where available, we fixed model parameter values from previous studies (Methods and Table 1). We estimated the remaining parameters by using non-linear mixed effects modelling to simultaneously fit viral load data (Fig 2) and bNAb concentrations (Fig 3) in the responder macaques and viral load data in the untreated macaques (Fig 4). The model provided good fits to the data, yielding population-level parameter estimates (Table 2) and estimates yielding best-fits to individual macaques (S1 and S2 Tables).

**Table 1:** Model parameters fixed from previous studies.

Parameter	Meaning	Value	Units	Citation
*d*_*T*_	Per capita death rate of uninfected CD4^+^ T cells	0.01	day^-1^	Ref. [18]
*d*_*I*_	Per capita death rate of productively infected CD4^+^ T cells	0.5	day^-1^	Ref. [18]
*c*	Viral clearance rate	38	day^-1^	Refs. [22, 32, 33]
*k*_*E*_	Per capita proliferation rate of effectors	0.1	day^-1^	Refs. [34, 35]
$q_{c}^{*}$	Half-maximal Hill function threshold for exhaustion	0.5	−	Ref. [19]
*n*	Hill coefficient for exhaustion	4	−	Ref. [19]
*d*_*q*_	Exhaustion reversal rate constant	0.1	day^-1^	Ref. [19]
$A_{1}^{dose}$	Estimated dose of the bNAb 3BNC117	65000^#^	*μ*g	Ref. [10]
$A_{2}^{dose}$	Estimated dose of the bNAb 10-1074	65000^#^	*μ*g	Ref. [10]
*ϵ*_*RTI*_	Efficacy of ART (reverse transcriptase inhibitor)	0.9	−	Ref. [18, 36, 37]
*ϵ*_*PI*_	Efficacy of ART (protease inhibitor)	0.95	−	Ref. [18, 36, 37]

^#^ Nishimura *et al*. administer 10 mg/kg of bNAbs to the macaques, and we assume an average body weight of 6.5 kg.

**Table 2:** Population parameter estimates (*μ*, *σ*) estimated by simultaneously fitting our model (Eqs 13–20) to data of *V*, *A*_1_ and *A*_2_ from untreated macaques and responders (Methods). Standard errors (SE) are shown in brackets.

Parameter	Meaning	*μ*	*σ*	Units of *μ*
*V*(0)	Initial plasma viral load	150 (179)	4.3 (1.2)	RNA copies mL^-1^
*ω*_1_	Delay in 3BNC117 action	2.1 (4.4)	0.3 (1.9)	days
*ω*_2_	Delay in 10-1074 action	1.1 (0.8)	0.1 (2.2)	days
*η*_1_	Clearance rate of 3BNC117	0.08 (0.01)	0.6 (0.1)	day^-1^
*η*_2_	Clearance rate of 10-1074	0.11 (0.02)	0.7 (0.2)	day^-1^
*Vol*_1_	3BNC117-specific distribution volume	324 (176)	1.1 (0.2)	mL
*Vol*_2_	10-1074-specific distribution volume	678 (126)	0.5 (0.2)	mL
*k*_1_	Maximal 3BNC117 efficacy	31 (86)	1.3 (1.0)	day^-1^
*k*_2_	Maximal 10-1074 efficacy	402 (340)	1.3 (0.5)	day^-1^
*K*	Half-maximal Hill function threshold for bNAb efficacy	122 (215)	0.9 (0.7)	*μ*g mL^-1^
*β*	Infectivity	1 × 10^−8^ (0.3 × 10^−8^)	0.1 (0.1)	RNA copies^-1^ mL day^-1^
p*	Burst size	6.6 × 10^9^ (2.1 × 10^9^)	0.2 (0.1)	RNA copies mL^-1^ day^-1^
m*	Killing rate of infected cells by effectors	10.7 (1.8)	0.1 (0.1)	day^-1^
*d*_*E*_	Per capita death rate of effectors	0.01 (0.002)	0.3 (0.2)	day^-1^
ϕ*	Half-maximal Hill function threshold for antigen sensitivity	3.7 × 10^−5^ (1.9 × 10^−5^)	1.0 (0.5)	-
*ξ*	Maximal rate of effector exhaustion	0.3 (0.1)	0.4 (0.1)	day^-1^
f*	Factor defining enhanced Ag presentation due to bNAbs	9 × 10^−10^ (24 × 10^−10^)	3.0 (1.9)	RNA copies^-1^ mL day

The fit-diagnostics we obtained indicated the robustness of the fitting procedure and parameter estimation. The population means (S1 Fig) and standard deviations (S2 Fig), the residual error parameters (S3 Fig), and the Akaike information criterion (AIC) (S4 Fig) all converged in our fits. Further, the distributions of the various parameters obtained from sampling agreed well with their theoretical distributions (S5 Fig). We performed formal sensitivity analysis of the model predictions to the variations in the parameter estimates (S6 Fig). We also verified that the population parameters compared well with previous estimates wherever available (Methods), giving us further confidence in the fits.

We ensured that the model contained the complexity essential to quantitatively capture the effect of bNAbs on the *in vivo* viral dynamics; model variants incorporating fewer details or features yielded worse fits or AIC (Table 3; Methods; S7–S14 Figs, S3–S9 Tables).

**Table 3:** Summary table comparing the Akaike information criterion (AIC) of the main model with model variants.

Model variant	AIC	Comments	Figure(s)	Table(s)
1. Basic virus dynamics model without an explicit effector response (Eqs 25–28)	4170	Model fits could not capture viral load data	S9 Fig	S4 Table
2. Model of post-ART control (Eqs 31–35)	4154	Model fits could not capture viral load data	S10 Fig	S5 Table
3. Hill coefficient, *n* = 3	4068	Model fits do not consistently capture reestablishment of control after effector depletion (see macaques MVJ and DEWP)	S11 Fig	S6 Table
4. Hill coefficient, *n* = 1	4223	Model fits could not capture viral load data	S12 Fig	S7 Table
5. Main model (Eqs 13–20)	3998		Figs 2, 3 and 4	Tables 1 and 2, S1 and S2 Tables
6. No enhanced antigen clearance by bNAbs (no *AV* term in Eq 15)	4152	Model fits could not capture viral load data	S13 Fig	S8 Table
7. No enhanced antigen uptake and effector elicitation by bNAbs (no f*AV term in Eq 16)	4008	Model fits do not capture the rebound viremic peak (after bNAb clearance) as well as the main model	S14 Fig	S9 Table
8. The effector activation and exhaustion thresholds are not same (*ϕ*_1_ ≠ *ϕ*_2_)	4078	Model fits did not capture viral load data better than the main model with *ϕ*_1_ = *ϕ*_2_ = *ϕ*	−	−
9. Varying the rate at which effectors proliferate (*k*_*E*_ in Eq 16)	4010	Model fits did not capture viral load data better than the main model with fixed *k*_*E*_ = 0.1 day^-1^	S8 Fig	S3 Table

The viral dynamics during and post bNAb therapy was complex and could be divided into the following phases (Fig 2). Viremia dropped post the acute infection peak to undetectable levels (< 10^2^ copies/mL), due to bNAb administration, where it remained until the administered bNAb level in circulation declined to a point where its ability to control viremia was lost (Phase I in Fig 2). Viremia then resurged to an elevated level (∼10^5^ copies/mL), well above the acute infection peak (< 10^4^ copies/mL), but subsequently declined spontaneously to a low (10^2^−10^3^ copies/mL) level (Phase II), where it remained for the rest of the duration of follow-up (>3 years; Phase III). The approach to steady state was a slowly dampened oscillation, similar to that seen in previous models of HIV-1 infection that considered an effector response [29–31].

Following the administration of anti-CD8 Abs, several months after the establishment of control, viremia immediately resurged to high levels again, but was eventually controlled (Phase IV) in 5 of 6 macaques thus treated (Fig 2). One macaque (DFIK) lost control following the administration of anti-CD8 Abs and reached a high viral load set-point, akin to untreated macaques.

The ability to describe these complex viral load changes during and post bNAb therapy quantitatively gave us confidence in our model. We applied our model next to elucidate the mechanistic origins of the observed long-term viremic control.

### Viremic control is a distinct outcome of infection

**Fig 5:**
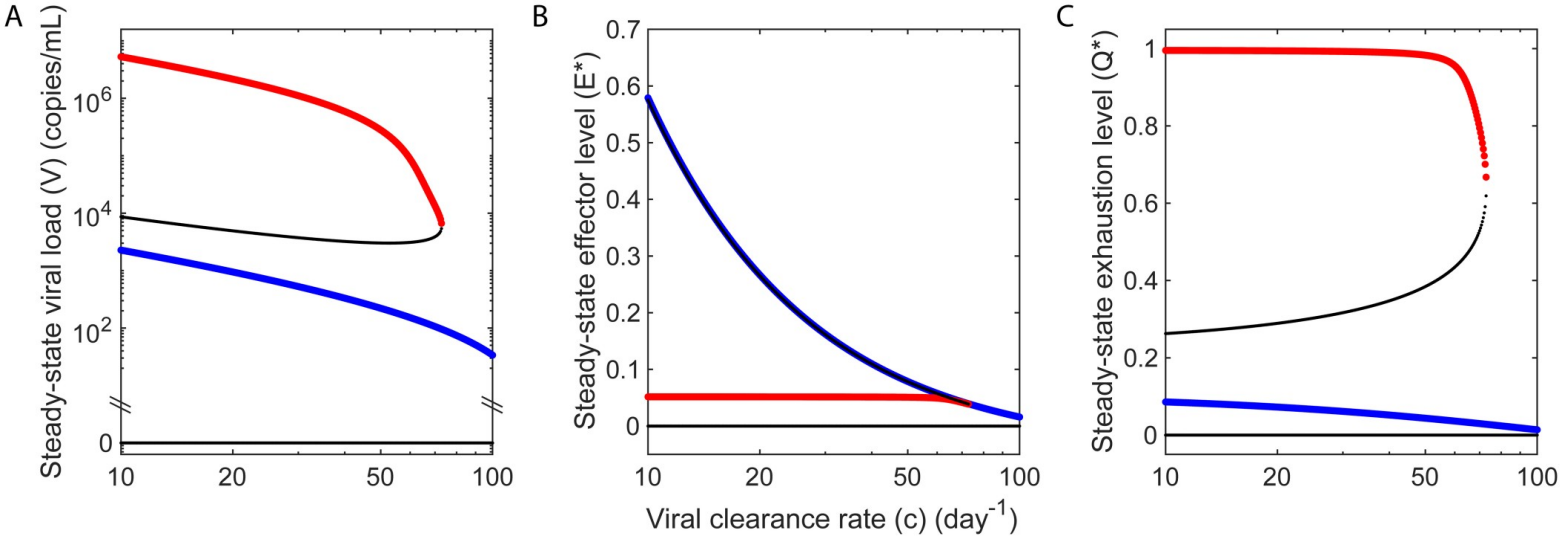
Bifurcation diagrams indicating the two distinct outcomes of progressive disease and viremic control. Model calculations of the steady state (**a**) viral load, (**b**) normalized effector level (E* = *Ed*_*E*_/*ρ*), and (**c**) normalized level of exhaustion (Q* = *Qd*_*q*_/*κ*), obtained by varying the viral clearance rate. The stable states of progressive disease and viremic control are shown in red and blue, respectively. Thin black lines represent unstable steady states. In (**b**), the intermediate unstable state lies close to the state of viremic control. The steady states are separated by other state variables, including the level of exhaustion, evident in (**c**). Parameter values used are in Tables 1 and 2. Bifurcation diagrams for other parameters are shown in S15 Fig.

We hypothesized, based on the two outcomes, progressive disease versus viremic control, achieved following bNAb therapy as well as CD8 depletion, that the lasting control observed with early bNAb therapy was a distinct steady state of the system. This hypothesis of bistability, or the existence of two distinct stable steady states, is consistent with a previous analysis of post-treatment control with ART [18] and may be a general feature of the interactions between antigen and effector cells [20]. Indeed, solving our model equations (Methods), we identified two underlying stable steady states (Fig 5). The first had high viremia, relatively high levels of effector exhaustion and relatively weak effector function, marking chronic infection leading to progressive disease. The second had low viremia, relatively low levels of effector exhaustion and relatively strong effector function, indicating lasting control. These states existed over wide ranges of parameter values (S15 Fig).

To indicate the two steady states for given parameter values, we use red and blue filled squares, respectively, in all the figures. The two stable states were separated by an unstable steady state of intermediate viremia, and intermediate effector stimulation and/or exhaustion levels. In addition, an uninfected steady state existed that was also unstable.

### Early, short-term bNAb therapy switches the outcome to viremic control via pleiotropic effects

**Fig 6:**
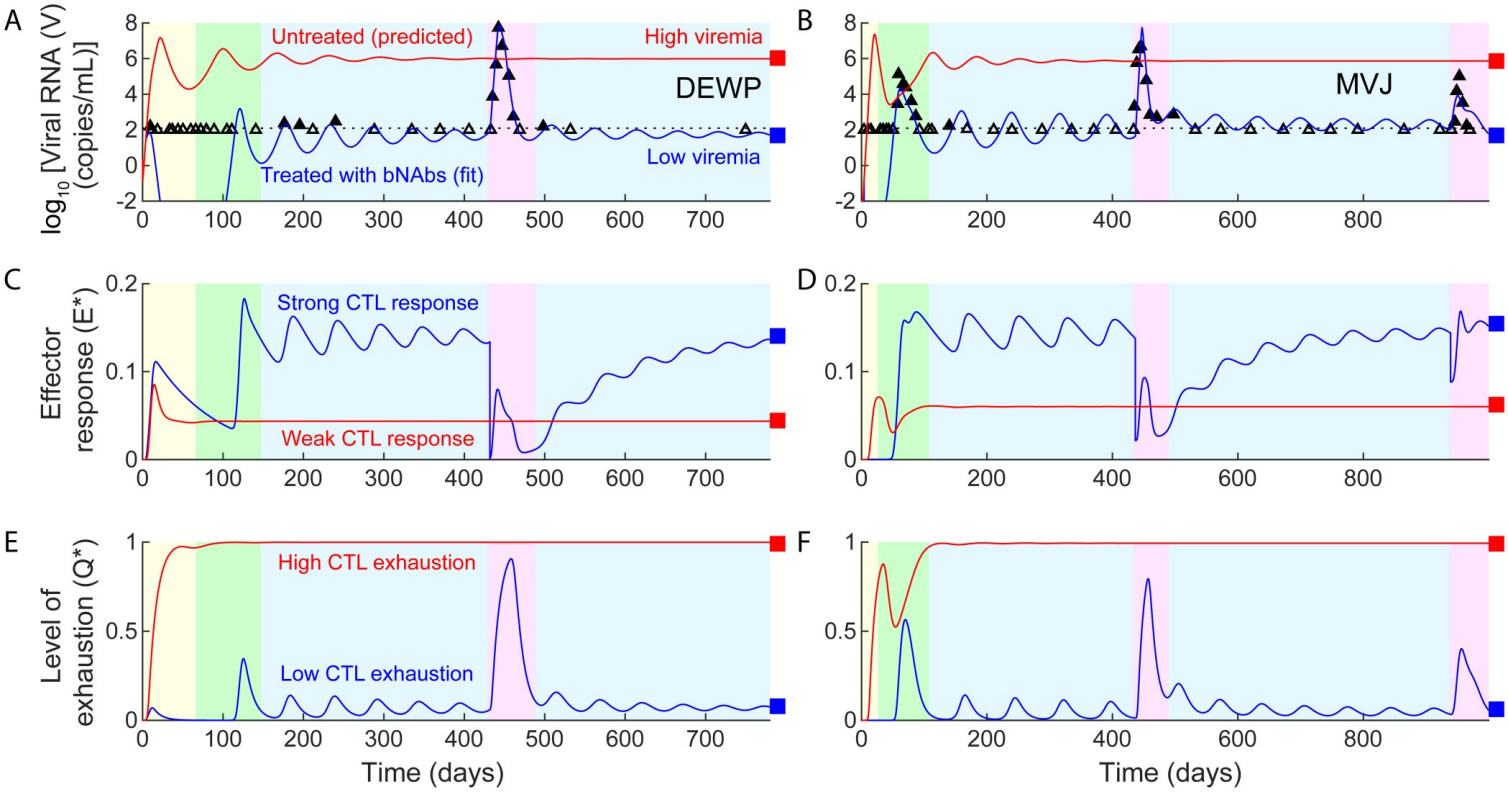
Early bNAb therapy induces a switch to viremic control. (**a,b**) Model fits (blue lines) to viral load data [10] (symbols) from the responder macaques DEWP and MVJ, representative of macaques displaying minimal or substantial viral load rebound post therapy, respectively. (The parameter values used are in Table 1 and S1 Table). The corresponding dynamics of (**c,d**) the effector response and (**e,f**) the level of effector exhaustion. The phases are color coded as in Fig 2. Red lines in all panels indicate model predictions with the same parameter values but in the absence of bNAb therapy. Our model predicts thus that bNAb therapy switches disease dynamics from reaching the high viremic, disease progressive state to the state of viremic control. Black dotted lines in (**a,b**) indicate the viral load detection limit (100 RNA copies/mL).

When a new infection occurs, viremia rises significantly during the acute infection phase and achieves high levels typically before an adaptive effector response is mounted [38]. The effector response thus lags and is dominated by the virus. If left untreated, the high viremic state, leading to progressive disease, is realized, as observed with untreated macaques challenged with SHIV [39, 40]. Our model predictions are consistent with these observations (Fig 4; red lines in Figs 2 and 6). They show that following the initial transients, a high viremia, a high level of exhaustion, and a weak effector response result.

Our model also predicts that early bNAb therapy drove the infection to the state of lasting viremic control (blue lines in Fig 6), in agreement with the observations in Nishimura *et al*. [10]. Further, our model predicts that pleiotropic effects of bNAbs were involved in orchestrating this transition: During acute infection, bNAbs suppressed the viremic peak by enhancing viral clearance. The lower viremia would limit effector exhaustion. Further, bNAbs would increase antigen presentation and effector stimulation. When the level of the administered bNAbs in circulation is diminished, viremia would resurge but in the presence of a primed effector pool, which would be larger and/or less severely exhausted than in untreated macaques (Fig 6c–6f). The primed effector population was able to control the infection in our predictions. Further, based on our predictions, when the effector response becomes significant before bNAb clearance, viremia would rise minimally post bNAb therapy, as observed with the macaque DEWP (Fig 6, left). When the effector response is weaker, viremia would resurge post bNAb therapy, but the limited effector exhaustion together with increased effector stimulation from the residual bNAbs would trigger a strong enough effector response to reassert control, akin to the observations with the macaque MVJ (Fig 6, right).

**Fig 7:**
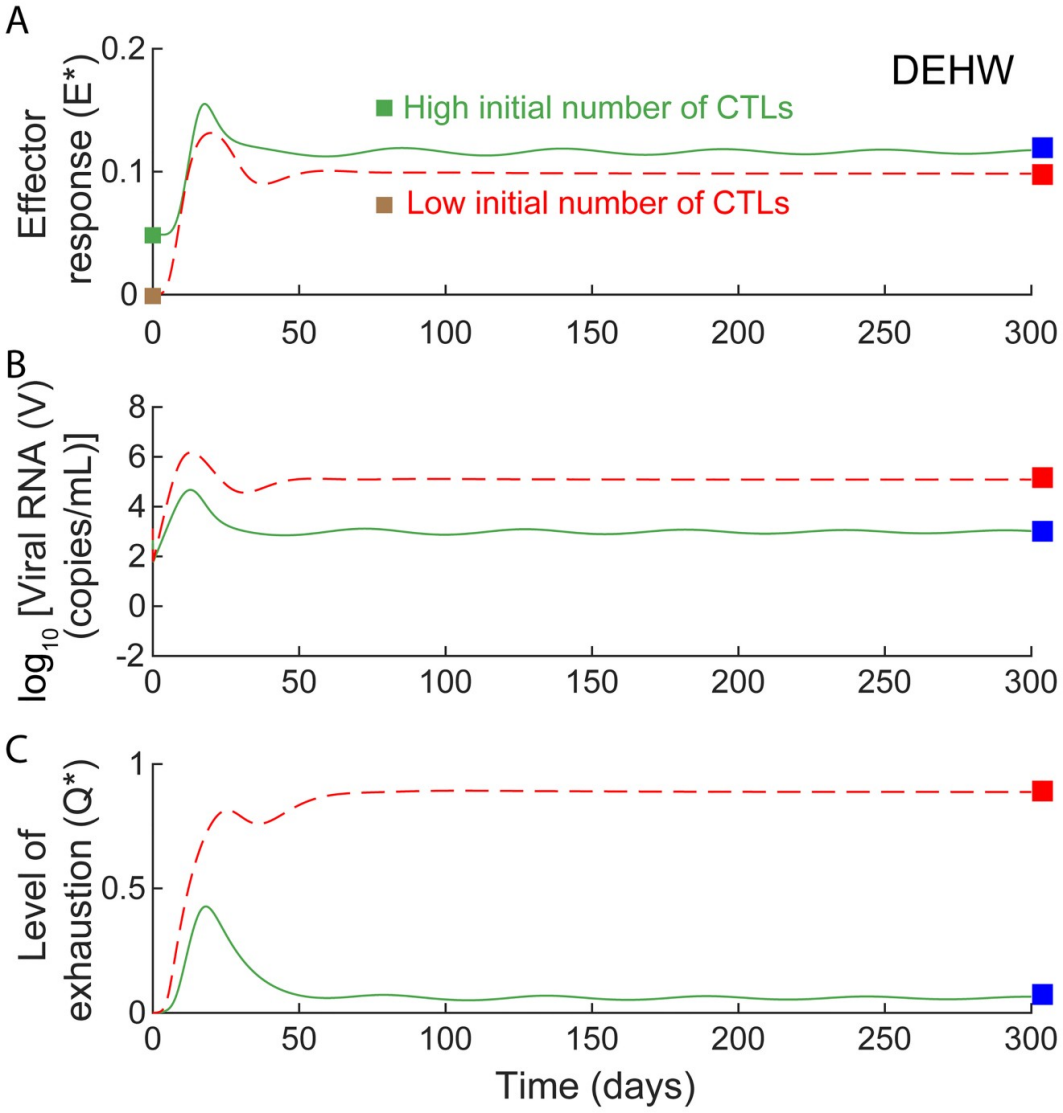
Model predictions of untreated infection dynamics with higher initial effector pool. Model predictions of effector, viral, and exhaustion dynamics for untreated infection show control with high (green) and progression with low (red) initial effector numbers (E*(0)). All other parameters are the same as those that yield the best-fit to the macaque DEHW (S1 Table).

The rise in viremia post treatment resembles an infection under conditions where the effector population is primed and thus has a head start over the virus. Indeed, our model calculations based on the hypothetical scenario where a higher effector population existed at the time of viral challenge showed spontaneous control (Fig 7).

Similarly, our model predicts that suppression of effectors due to anti-CD8 Abs up to a threshold after the establishment of control allowed resurgence in viremia but restored control, whereas suppression beyond the threshold sacrificed control and drove infection to the former, high viremic fate (Fig 2), explaining the difference between the controller macaques who reasserted viremic control following the transient rise in viremia upon the administration of anti-CD8 Abs and the one controller macaque (DFIK) that failed to reassert control and transitioned to the high viremic state [10]. Note that the threshold can vary across macaques and corresponds, in our model, to the unstable boundary separating the stable states of viremic control and progressive disease (Fig 5).

When we repeated our analysis without either enhanced clearance of virus or upregulation of antigen presentation by bNAbs, our model failed to capture the observed viral load data robustly (S13 and S14 Figs, and S8 and S9 Tables). Specifically, neglecting bNAb-induced enhancement of viral clearance did not fit the data well (S13 Fig), whereas neglecting bNAb-induced upregulation of antigen presentation yielded fits with a higher value of the AIC (S14 Fig, Table 3). Our analysis suggests, thus, that the rapid clearance of the virus, which lowered viremia and reversed effector exhaustion, together with increased antigen uptake, which led to enhanced effector stimulation, resulted in the lasting control of viremia elicited by early, short-term bNAb therapy.

### bNAbs have an advantage over ART

**Fig 8:**
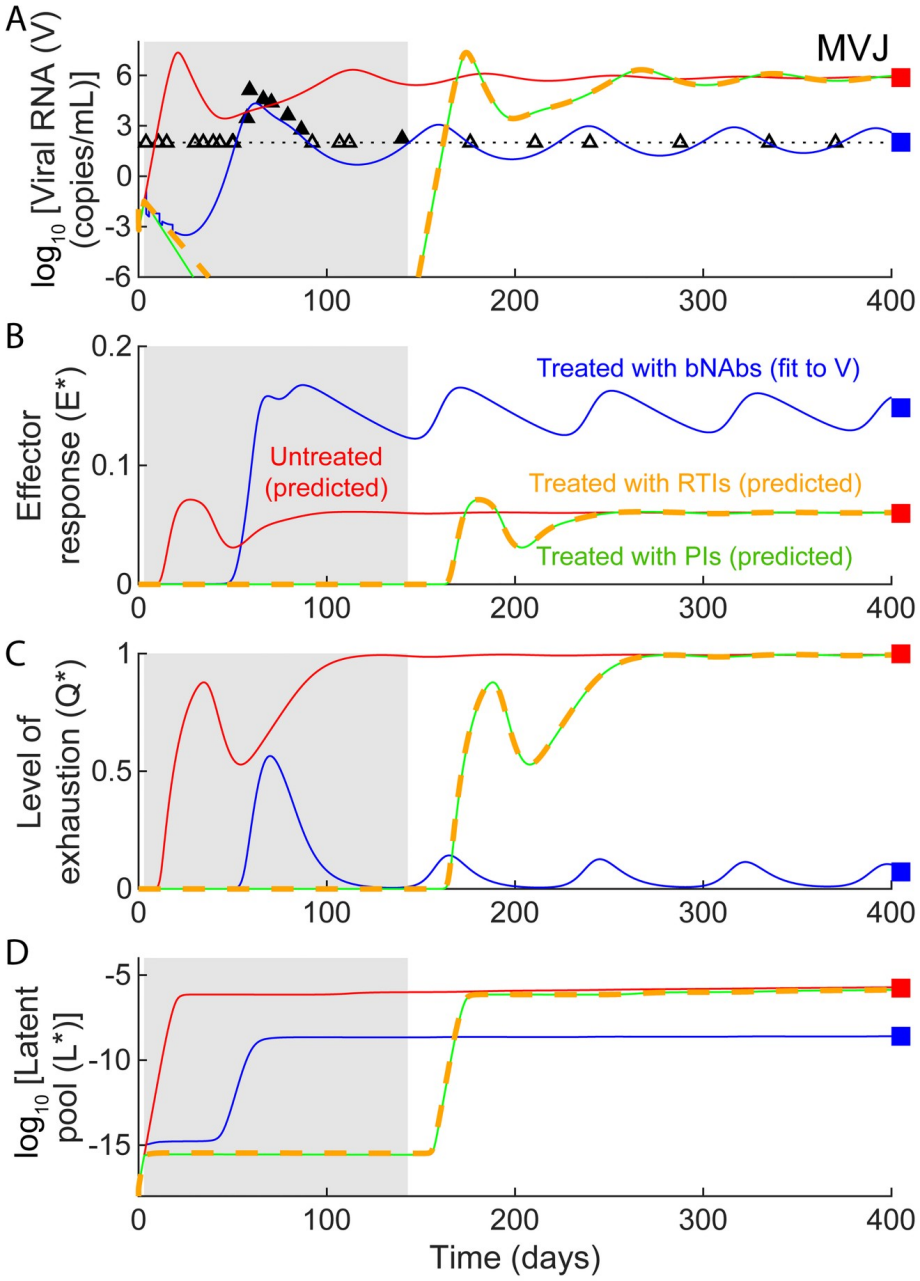
bNAbs succeed whereas ART fails to establish long-term viremic control. Simulated dynamics of (**a**) viral load, (**b**) effector response, (**c**) effector exhaustion, and (**d**) the latent reservoir, are shown with ART (reverse transcriptase inhibitors—green solid lines, protease inhibitors—orange dashed lines; Methods) in comparison with the corresponding dynamics without treatment (red lines) and with bNAb treatment (blue lines) using parameters that capture *in vivo* data for the macaque MVJ (symbols). (Parameter values used are in Table 1 and S1 Table). The duration of ART is shown as a gray region. The black dotted lines in (**a**) indicates the viral load detection limit (100 RNA copies/mL).

If ART were used instead of bNAbs, for a duration equivalent to the time over which the administered bNAbs were in circulation, viral load quickly became undetectable during treatment in our model but rebounded to high levels post treatment (Fig 8), consistent with the 3 macaques administered ART in Nishimura *et al*. [10]. While both bNAb therapy and ART limited viremia (Figs 6a, 6b and 8a) and would thus prevent effector exhaustion (Figs 6e, 6f and 8c), bNAb therapy can additionally increase antigen presentation and effector stimulation (Figs 6c, 6d and 8b). The resurgence in viremia post the cessation of ART would then be akin to *de novo* infection, which typically reaches the high viremic fate. Only in the rare individuals in whom the latent reservoir is sufficiently small is this rise in viremia small enough for the effector population to catch up and establish control, explaining the critical role of the size of the latent reservoir in post treatment control with ART [2, 18, 41]. The primed effector population following bNAb therapy, in contrast, drove the system to viremic control in our model. Consquently, the dynamics was less sensitive to the size of the latent reservoir (S7 Fig). Because of their pleiotropic effects, bNAbs would thus have a significant advantage over ART, explaining their much higher success rate in achieving lasting control.

## Discussion

The recent success of passive immunization with bNAbs in eliciting functional cure of HIV-1 infection can potentially revolutionize HIV-1 care [10–17]. In this study, using mathematical modelling and analysis of *in vivo* data, we present a dynamical systems view of the infection that offers an explanation of how early exposure to bNAbs induces lasting viremic control and does so better than ART.

Our analysis suggests that progressive disease and viremic control are states ‘intrinsic’ to the system. Interventions, with ART or bNAbs, can serve to alter the propensity with which the states are realized. In untreated infection, rapid viral growth can induce significant effector exhaustion and lead typically to progressive disease. ART suppresses viremia and would therefore at least in part reverse exhaustion. (Recent studies have argued that CTL exhaustion may not be fully reversible [42].) However, because successful ART can completely block viral replication, the absence of antigenic stimulation could eventually cause the activated effector population to fade. When viremia rises post treatment, if the activated effector population has faded significantly, the dynamics would mimic *de novo* infection leading to progressive disease. Indeed, post-ART controllers have been found to have low CTL activation levels [8]. The resurgence of viremia post ART is due to the reactivation of latently infected cells. When the latent pool is small, this reactivation may lead to low viremia, which can stimulate effectors and culminate in viremic control. Post-ART controllers were indeed found to have small latent reservoir sizes [8]. A model developed to explain post-ART control accordingly found a critical dependence on the size of the latent reservoir [18]. In contrast, bNAbs can additionally stimulate effectors by enhancing antigen presentation, a phenomenon first identified with cancers and termed the ‘vaccinal effect’ [43]. Our model was most consistent with data when this effect was incorporated. When viremia rises post bNAb therapy, it would encounter a primed effector population, which can drive the system to viremic control. Unless intense bNAb therapy completely halts active viral replication, akin to ART, and limits the primed CTL pool, the establishment of control is less likely to be sensitive to the latent reservoir size. Indeed, our model fit the macaque data without considering latently infected cells. bNAbs would therefore achieve lasting control in a far greater percentage of the population treated than ART.

Previous studies either examined the steady states alone [18] or short-term viral load changes following exposure to bNAbs [24]. Going beyond, our study captures the entire time course of viral load changes leading to lasting control, resulting in a more comprehensive understanding of functional cure. Our model predicts that through pleiotropic effects, bNAbs tilt the balance of the competing interactions between the virus and effectors in favour of effectors. It follows that other strategies that similarly tilt the balance may also elicit functional cure. For instance, immune checkpoint inhibitors can prevent effector exhaustion during infection [44]. Alternatively, effector cells may be adoptively transferred, a strategy that showed promise in a recent macaque study [45]. Effector cells can also be stimulated using vaccines [46, 47]. Our model predicts that a primed effector population at the time of viral challenge may prevent the infection from reaching the high viremic, progressive state and drive it instead to lasting viremic control, indicating a potentially favourable outcome of preventive T cell vaccines. Stimulating effectors using vaccines has been shown to suppress viral replication and lower the set-point viremia [47, 48], but several additional design challenges must be overcome, including the need to stimulate effectors early enough and, interestingly, prevent effector exhaustion due to the vaccine [49], for the successful deployment of T cell vaccines [50]. Further, such strategies may not work in children, where bNAbs may elicit control without effector responses, possibly due to the inadequately developed immune system [51].

Following the observation of Nishimura *et al*. [10] that there was no significant generation of anti-gp120 Abs in controllers, we ignored the ability of bNAbs to upregulate the humoral response [52, 53]. Our study is thus conservative in its assessment of the influence of bNAbs. We did not consider data from the three non-responder macaques, where functional cure was not established and CD4^+^ T cell counts gradually declined [10]. Describing the entire course of HIV infection from the acute phase to AIDS using a single model has been a long-standing challenge [54]. One possibility behind the failure of bNAb therapy in the non-responders is that the bNAb therapy induced rapid viral load decline, limiting effector stimulation, and thus behaved like ART in the non-responders [10]. Alternatively, the non-responders may correspond to parameter settings in our model that did not admit bistability, a possibility also with those who failed to achieve post-ART control [18]. Note that the determinants of post-ART control are yet to be established [8, 9]. Finally, we did not consider viral evolution and resistance to bNAbs, recognizing that most controllers in Nishimura *et al*. [10] saw no resistance. Future studies with other combinations of bNAbs may determine whether resistance must be accounted for in defining optimal bNAb treatments, especially for use during chronic infection where viral diversity is likely to be large [55].

In summary, our study presents the first quantitative description of viral dynamics following passive immunization with HIV-1 bNAbs; captures *in vivo* data; elucidates the mechanisms with which early, short-term bNAb therapy establishes lasting viremic control of SHIV infection; explains the advantages of bNAbs over ART; and suggests alternative avenues to induce functional cure of HIV-1 infection.

## Methods

We first present the model (Fig 1) that best fit the *in vivo* macaque data (Figs 2–4). We next describe its solution procedure, data fitting and parameter estimation. At the end, we outline the rational strategy that we employed to arrive at the model by gradually adding complexity to the basic model of viral dyanmics and assessing the resulting model at each stage.

### Model equations

The following equations described the viral dynamics depicted schematically in Fig 1.

$$\begin{array}{ccc} \frac{dT}{dt} & = & \lambda_{T}-\beta VT-d_{T}T \end{array}$$ (1)

$$\begin{array}{ccc} \frac{dI}{dt} & = & \beta VT-(d_{I}+mE)I \end{array}$$ (2)

$$\begin{array}{ccc} \frac{dV}{dt} & = & pI-(c+A)V \end{array}$$ (3)

$$\begin{array}{ccc} \frac{dE}{dt} & = & \frac{\left(\rho +k_{E}E)(I+fAV\right)}{\left(\phi_{1}+I+fAV\right)}-(\frac{\xi Q^{n}}{{\left(q_{c}\right)}^{n}+Q^{n}}+d_{E})E \end{array}$$ (4)

$$\begin{array}{ccc} \frac{dQ}{dt} & = & \frac{\kappa I}{\phi_{2}+I}-d_{q}Q \end{array}$$ (5)

$$\begin{array}{ccc} A_{1} & = & \left(\frac{A_{1}^{dose}}{Vol_{1}})\sum_{i=1}^{3}e^{-\eta_{1}(t-(\tau_{i}+\omega_{1}))}\;H(t-(\tau_{i}+\omega_{1})\right) \end{array}$$ (6)

$$\begin{array}{ccc} A_{2} & = & \left(\frac{A_{2}^{dose}}{Vol_{2}})\sum_{i=1}^{3}e^{-\eta_{2}(t-(\tau_{i}+\omega_{2}))}\;H(t-(\tau_{i}+\omega_{2})\right) \end{array}$$ (7)

$$\begin{array}{ccc} A & = & \frac{k_{1}A_{1}+k_{2}A_{2}}{K+A_{1}+A_{2}} \end{array}$$ (8)

Here, uninfected target CD4^+^ T cells, *T*, are produced at the rate λ_*T*_, die at the per capita rate *d*_*T*_, and are infected by virions, *V*, with the second order rate constant *β*, yielding productively-infected cells, *I*. Cells *I* die at the per capita rate *d*_*I*_ due to viral cytopathicity and are killed by effector cells, *E*, with the second order rate constant *m*. Cells *I* produce free virions at the rate *p* per cell. The virions are cleared in the absence of administered bNAbs with the rate constant *c*. Administered bNAbs are assumed to induce a net enhancement of the viral clearance rate by a time-dependent amount, *A*, which is a saturable function of the instantaneous serum concentration of the two bNAbs, 3BNC117 (*A*_1_) and 10-1074 (*A*_2_). The functional form mimics the 2D Hill equation derived recently to describe the effect of drug combinations [56, 57] and has been used to quantify combinations of Abs and virus entry inhibitors [57, 58], expected to be similar to the combination of bNAbs targeting non-overlapping sites on the HIV-1 envelope used here [10]. The net enhancement of viral clearance combines the direct effect on viral clearance by bNAbs [22] as well as the reduction in infectivity, *β*, due to viral neutralization by the bNAbs [59]. By preventing new infections, virus neutralization lowers the number of infected cells and hence overall viral production. Because viral production and clearance are rapid compared to other processes [60], the effect of lower viral production on viral dynamics is indistinguishable through viral load measurements from the effect of enhanced viral clearance. Indeed, the pseudo-steady state approximation applied to Eq (3) yields *V* ≊ *pI*/(*c* + *A*), which when substituted into Eqs (1) and (2) results in net infectivities of *βp*/(*c* + *A*) and *βp*/*c* with and without bNAbs, respectively, thus amounting to a reduction in *β* by the factor (*c* + *A*)/*c*. Conversely, a reduction in *β* can be subsumed into an effect on *c*.

Administered bNAbs also bind to virions and increase antigen uptake by antigen presenting cells (APCs), which in turn would increase stimulation of effectors. If we define *P* as the population of activated APCs, which can thus stimulate effectors, then we may write *dP*/*dt* = *γI* + *νAV* − *d*_*P*_*P*, where *γ* is the rate constant of APC stimulation by infected cells, *d*_*P*_ their per capita death rate, and *ν* the fractional rate of opsonized virions cleared (AV) that is taken up by APCs. We assume naïve APCs to be in large excess. Assuming pseudo steady state yields *P* = (*γI* + *νAV*)/*d*_*P*_. Finally, letting effector cell stimulation be a saturable function of *P* and simplifying yields the expression in Eq (4) for effector stimulation by antigen. In effect, the total serum antigen available for presentation to effectors, *I* + *fAV*, stimulates immune cells such as naïve CD8^+^ T cells [19, 61], NK cells [62], and others, assumed not to be limiting, into antiviral effectors, *E*, with the maximal rate, *ρ*, and the half-maximal antigen-sensitivity parameter, *ϕ*_1_. (Here, *f* = *ν*/*γ*). We note that *E* is a composite effector pool consisting of SHIV-specific CTLs, NK cells, and other effectors [10, 18, 63]. The saturation of the stimulation with increasing levels of total serum antigen is reflective of limitations in cellular interaction processes, such as the time to find interacting partners, the duration of each interaction, and cytokine/chemokine signalling [62, 64–66]. Saturating forms are argued to be more realistic than mass-action forms [65] and follow from kinetic considerations of these interactions under the total quasi-steady state approximation [66]. Following activation, effectors such as CTLs and NK cells enter a proliferation program that does not require antigen but can be modulated by antigen, cytokines and other stimulatory and co-stimulatory signals [62, 67–70]. Accordingly, we let effectors proliferate [71] with the rate constant *k*_*E*_, and, for simplicity, subject to the same saturating dependence on antigen as activation. Effectors suffer exhaustion [19, 72] at the maximal rate *ξ*, with the half-maximal parameter *q*_*c*_ and Hill coefficient *n* = 4. The level of exhaustion, *Q*, representative of cumulative antigen exposure, increases with antigen level at the maximal rate *κ*, and with the half-maximal parameter *ϕ*_2_. Exhaustion is reversed with the rate constant *d*_*q*_. Effectors are lost at the per capita rate *d*_*E*_. We note that *E* is a measure of the effective effector pool, consisting of activated NK cells and SHIV-specific CTLs, and factoring in the overall level of exhaustion, *Q*. *E* is thus not to be viewed as a cell count. Similarly, *Q* is not a measure of the pool of exhausted cells. Our model of effector stimulation and exhaustion mimics an earlier study [19].

To mimic the dosing protocol in Nishimura *et al*. [10], we let the serum concentration levels of the bNAbs, *A*_1_ and *A*_2_, rise by the extent $A_{1}^{dose}/Vol_{1}$ and $A_{2}^{dose}/Vol_{2}$, immediately upon the administration of a bNAb dose, and decline exponentially with the rate constants *η*_1_ and *η*_2_, respectively. Here, $A_{1}^{dose}$ and $A_{2}^{dose}$ correspond to the dosages of the two bnAbs, and *Vol*_1_ and *Vol*_2_ correspond to the bNAb-specific volumes of distribution. The three doses were administered on days *τ*_1_ = 3, *τ*_2_ = 10, and *τ*_3_ = 17, respectively. Additionally, we allowed a delay in bNAb action, *ω*_1_ and *ω*_2_, following each dose, to account for any pharmacological effects within individual macaques. The Heaviside function, *H*(*t* < (*τ*_*i*_ + *ω*_*j*_)) = 0 and *H*(*t* ≥ (*τ*_*i*_ + *ω*_*j*_)) = 1, accounts for bNAb dynamics based on these dosing times. The maximal efficacies of the respective bNAbs, achieved when they are in excess, are set to *k*_1_ and *k*_2_, respectively, and their half-maximal efficacy is defined by the Hill function threshold *K*.

We next compare the influence of bNAbs with that of ART. We let reverse transcriptase inhibitors (RTIs), which prevent cells from being productively infected by the virus, reduce *β* by a factor 1 − *ϵ*_*RTI*_, where *ϵ*_*RTI*_ = 0.9 is the efficacy of an all-RTI drug combination [18, 36, 37]. (*ϵ*_*RTI*_ = 1.0 implies a 100% effective inhibitor.) We let protease inhibitors, which prevent the maturation of immature virions into infectious viruses, partition the viral population into two sub-populations, *V*_*I*_ and *V*_*NI*_, where *V*_*I*_ are infectious virions and *V*_*NI*_ are immature, non-infectious virions produced due to the action of the protease inhibitors with an efficacy *ϵ*_*PI*_ = 0.95 [18, 36, 37]. The virus dynamics can now be written as:

$$\begin{array}{ccc} \frac{dV_{I}}{dt} & = & p(1-\epsilon_{PI})I-(c+A)V_{I} \end{array}$$ (9)

$$\begin{array}{ccc} \frac{dV_{PI}}{dt} & = & p\epsilon_{PI}\;I-(c+A)V_{PI} \end{array}$$ (10)

where the total viral load, *V*, is *V*_*I*_ + *V*_*NI*_. Because the size of the latent reservoir was shown to be important for the establishment of post-ART control [2, 18, 41], we added the latent reservoir dynamics to our model above, following a model of post-ART control [18]. The resulting equations and parameter values are discussed below in the ‘Model building strategy’ subsection. Model predictions with and without the latent pool showed little difference to our fitting (S7 Fig), which is expected because active replication is never fully halted in our fits, as opposed to the expectation during ART. Consequently, the latent reservoir assumes far greater significance with ART than bNAb therapy, allowing us to neglect the latent reservoir, except in our comparisons between bNAb therapy and ART (Fig 8).

Long after bNAbs were cleared from circulation, Nishimura *et al*. [10] administered anti-CD8*α* antibodies to some macaques, which resulted in the depletion of effectors such as CD8^+^ T, NK, NKT, and *γδ* T cells. Nishimura *et al*. [10] also administered anti-CD8*β* antibodies to some macaques, which presumably only depleted CD8^+^ T cells. To describe the resulting changes in viremia, we assumed that the depleting effects of anti-CD8*α* and anti-CD8*β* antibodies started at time points *θ*_*α*_ and *θ*_*β*_, respectively, at which points we reduced the effector populations by fractions *ζ*_*α*_ and *ζ*_*β*_. Further, we assumed that anti-CD8*α* antibodies neutralized all host effector functions, which we modelled by setting *m* = 0 for a duration *θ*_*m*_ representing the residence time of the depleting antibodies.

The procedures for solving the above model equations, parameter estimation, sensitivity analysis, and data fitting, are described next. All the data employed for fitting was obtained by digitizing the data published in Nishimura *et al*. [10] using the software Engauge digitizer, and is available as a supplementary excel file (S1 Data).

### Solution of model equations

For ease of solution and parameter estimation, we rescaled Eqs 1–8 in the main text using the quantities in Eqs 11 and 12 and obtained an equivalent model with fewer parameters (Eqs 13–20). bNAb concentrations, *A*_1_ and *A*_2_, and viral load, *V*, were not scaled because they were used for fitting data.

$$\begin{array}{ccc} T^{*} & = & T\frac{d_{T}}{\lambda_{T}},\;\;I^{*}=I\frac{d_{T}}{\lambda_{T}},\;\;E^{*}=E\frac{d_{E}}{\rho },\;\;Q^{*}=Q\frac{d_{q}}{\kappa } \end{array}$$ (11)

$$\begin{array}{ccc} p^{*} & = & p\frac{\lambda_{T}}{d_{T}},\;\;m^{*}=m\frac{\rho }{d_{E}},\;\;f^{*}=f\frac{d_{T}}{\lambda_{T}},\;\;\phi^{*}=\phi_{1}\frac{d_{T}}{\lambda_{T}}=\phi_{2}\frac{d_{T}}{\lambda_{T}},\;\;q_{c}^{*}=q_{c}\frac{d_{q}}{\kappa } \end{array}$$ (12)

$$\begin{array}{ccc} \frac{dT^{*}}{dt} & = & d_{T}(1-T^{*})-\beta VT^{*} \end{array}$$ (13)

$$\begin{array}{ccc} \frac{dI^{*}}{dt} & = & \beta VT^{*}-(d_{I}+m^{*}E^{*})I^{*} \end{array}$$ (14)

$$\begin{array}{ccc} \frac{dV}{dt} & = & p^{*}I^{*}-(c+A)V \end{array}$$ (15)

$$\begin{array}{ccc} \frac{dE^{*}}{dt} & = & \frac{\left(d_{E}+k_{E}E^{*})(I^{*}+f^{*}AV\right)}{\left(\phi^{*}+I^{*}+f^{*}AV\right)}-(\frac{\xi {\left(Q^{*}\right)}^{n}}{{\left(q_{c}^{*}\right)}^{n}+{\left(Q^{*}\right)}^{n}}+d_{E})E^{*} \end{array}$$ (16)

$$\begin{array}{ccc} \frac{dQ^{*}}{dt} & = & d_{q}(\frac{I^{*}}{\phi^{*}+I^{*}}-Q^{*}) \end{array}$$ (17)

$$\begin{array}{ccc} A_{1} & = & \left(\frac{A_{1}^{dose}}{Vol_{1}})\sum_{i=1}^{3}e^{-\eta_{1}(t-(\tau_{i}+\omega_{1}))}\;H(t-(\tau_{i}+\omega_{1})\right) \end{array}$$ (18)

$$\begin{array}{ccc} A_{2} & = & \left(\frac{A_{2}^{dose}}{Vol_{2}})\sum_{i=1}^{3}e^{-\eta_{2}(t-(\tau_{i}+\omega_{2}))}\;H(t-(\tau_{i}+\omega_{2})\right) \end{array}$$ (19)

$$\begin{array}{ccc} A & = & \frac{k_{1}A_{1}+k_{2}A_{2}}{K+A_{1}+A_{2}} \end{array}$$ (20)

The model equations were integrated using the initial conditions T*(0) = 1, I*(0) = E*(0) = Q*(0) = 0, $A_{1}(0)=\frac{A_{1}^{dose}}{Vol_{1}}$ and $A_{2}(0)=\frac{A_{2}^{dose}}{Vol_{2}}$. The initial viral load, *V*(0), varied across macaques and was estimated from fits.

### Parameter estimates, sensitivity analysis, and data fitting

The data available from Nishimura *et al*. [10] for fitting this model consists of longitudinal values of the plasma SHIV viral load (*V*) and serum bNAb concentrations (*A*_1_ and *A*_2_). For the above model, we performed formal identifiability analysis of the model parameters by employing the Exact Arithmetic Rank (EAR) implementation in Mathematica (IdentifiabilityAnalysis package [73]), assuming that measurements of *V*, *A*_1_ and *A*_2_ are perfect and continuous in time. The analysis revealed that upon fixing $A_{1}^{dose}$ and $A_{2}^{dose}$, whose values can be estimated from Nishimura *et al*. [10], all the other parameters in the model are fully identifiable from the data.

Wherever possible, we fixed model parameter values based on previously published estimates (Table 1). For instance, following Conway and Perelson [18], we fixed the death rate of target cells, *d*_*T*_ = 0.01 day^-1^. We let the death rate of infected cells due to viral cytopathicity be *d*_*I*_ = 0.5 day^-1^, which is ∼ 50% of the mean overall death rate of infected cells [64], and lies at the lower end of the range of the overall death rate, 0.5–1.7 day^-1^ for HIV-1 [74], with the rest attributed to effector killing. The viral clearance rate, *c*, was estimated to be in the range 9–36 day^-1^ for HIV-1 [18, 75] and in the range 38–302 day^-1^ for SIV [22, 32, 33]. For SHIV infection studied here, we chose a value of *c* = 38 day^-1^, in the intersection of the two ranges. To estimate the effector expansion rate, *k*_*E*_, we obtained fits with *k*_*E*_ adjustable, which yielded best-fit *k*_*E*_ ∼ 0.3 day^-1^ (S9 Fig and S3 Table). The model, however, had a higher value of the AIC than when *k*_*E*_ was fixed (Table 3). Here, we therefore chose a model with fixed *k*_*E*_ and set *k*_*E*_ = 0.1 day^-1^, a smaller value to reflect the average expansion rate of all effectors and not CTLs alone, with other effectors typically displaying a lower expansion rate than CTLs [18]). We set the Hill coefficient for exhaustion, *n* = 4, following studies that recognize underlying non-linearities [18, 19] and because smaller integral values of *n* did not yield good fits (see subsection ‘Model building strategies’ below). Further, we set *ϕ*_1_ = *ϕ*_2_ = *ϕ* following earlier studies [19] and because fitting them as separate variables yielded best-fit values that were close, *ϕ*_1_ ∼ 2.1 × 10^−5^ and *ϕ*_2_ ∼ 5.7 × 10^−5^, but with a higher AIC (Table 3).

We divided the remaining parameters into two sets, *ϑ* = {*β*, m*, p*, f*, ϕ*, *d*_*E*_, *ξ*, *V*(0), *Vol*_1_, *Vol*_2_, *k*_1_, *k*_2_, *K*} and *ϱ* = {*ω*_1_, *ω*_2_, *η*_1_, *η*_2_}, the former assumed to follow log-normal and the latter logit-normal distributions, respectively. To estimate these parameters, we employed a population-based fitting approach using non-linear mixed effects (NLME) models to jointly fit the log plasma viral load (both treated and untreated animals) and antibody concentrations of the ten responder macaques in Nishimura *et al*. [10] which do not exhibit consistent CD4^+^ decline: DEMR, MVJ, DEWP, DEWL, MAF, DFIK, DFKX, DFFX, DEHW, and DEBA.

Briefly, the parameters for all the individual macaques were assumed to be sampled from a common population distribution, and the aim of the fitting exercise was to obtain estimates for the mean and variance of this distribution for each parameter. For each macaque *i*, parameters *ϑ*_*i*_ were estimated assuming underlying log-normal distributions of the form:

$$\begin{array}{c} log(\vartheta_{i})=log\;\;\mu +\psi_{i} \end{array}$$ (21)

where *μ* is the set of the population means and *ψ*_*i*_ ∼ *N*(0, *σ*) are normally distributed ‘random effects’ whose variances are to be estimated. Similarly, parameters *ϱ_i_* for macaque *i* were estimated assuming underlying logit-normal distributions of the form:

$$\begin{array}{c} log(\frac{\varrho_{i}-\varrho_{i}^{min}}{\varrho_{i}^{max}-\varrho_{i}})=log(\frac{\mu -\varrho_{i}^{min}}{\varrho_{i}^{max}-\mu })+\psi_{i} \end{array}$$ (22)

where $\varrho_{i}^{min}$ and $\varrho_{i}^{max}$ are the minimum and maximum allowed values for parameters in *ϱ_i_*. The latter limits were set so that *ω*_1_, *ω*_2_ ∈ [0, 5], and *η*_1_, *η*_2_ ∈ [0.01 − 7] day^-1^, expected from the 1 week dosing interval employed. To account for measurement errors in observed viral loads and bNAb levels (*y*(*t*)), we defined a ‘combined error’ model such that *y*(*t*) is normally distributed around a true value (y*(*t*)) as described by the following equation:

$$\begin{array}{c} y\left(t\right)=y^{*}\left(t\right)+(a+b\;y^{*}\left(t\right))\;\psi_{y}\left(t\right),\;\psi_{y}∼N\left(0,1\right) \end{array}$$ (23)

where *a* is the constant error term and *b* is the error term proportional to the true value, y*(*t*).

All fitting except CD8 depletion was performed using Monolix software version 2019R1 (www.lixoft.eu), where the population estimates were obtained by maximum likelihood estimation. Untreated monkeys infected with the SHIV_AD8-EO_ viral strain consistently proceed towards high viremic chronic infection and immunodeficiency [39, 76, 77]. To recapitulate this behaviour, we simultaneously solved the model without bNAbs and ensured that the set point viral load attained values greater than 10^3.5^ copies/mL (enforced using the ‘censoring’ keyword during data input in Monolix). Parameter space exploration was carried out using the stochastic approximation using expectation maximization (SAEM) algorithm implemented in Monolix [78]. We simulated a large number of SAEM realizations (100–200) that succeeded in converging to consistent maximum-likelihood parameter estimates that were biologically realistic (S1–S3 Figs). The model AIC, estimated from the empirical log-likelihood (*LL*) obtained from an importance sampling Monte Carlo method (*AIC* = −2*LL* + 2*n*, with *n* the number of parameters estimated), also showed good convergence (S4 Fig). Empirically obtained individual parameter distributions were consistent and agreed well with theoretical distributions estimated by Eqs 21 and 22 (S5 Fig). Robust standard error estimation (Table 2) required a large number of iterations (> 10^5^), and was performed separately for the bNAb PK parameters and the rest.

Comparisons of some of the population parameter means with independent estimates from previous studies further confirmed that our parameter estimates were biologically realistic and validated our fitting procedure. For instance, the estimated population value of p* corresponds to a burst size of 3298 virions per infected cell (Table 2), consistent with current estimates of ∼10^4^ virions per cell [79] given a target cell production rate [18] of λ_*T*_ = 10^4^ cells mL^-1^ day^-1^. Similarly, the estimated infectivity, *β* = 10^−8^ RNA copies^−1^ mL day^−1^, is close to the previous estimate of 2.4 × 10^−8^ virion^−1^ mL day^−1^ [74]. Finally, we note that the enhanced clearance rate of virions, *c* + *A*, has a maximum of ∼145 day^-1^, which is ∼3.8-fold above the natural clearance rate, *c* = 38 day^-1^, consistent with the 3–4 fold increase in clearance rate due to bNAbs observed in other studies [22]. We note that while our fits quantitatively captured the overall influence of bNAbs, delineating the individual contributions of 3BNC117 and 10-1074 may require either more frequent measurements or similar studies with monotherapy; the standard error estimates of some of the parameters quantifying the individual bNAb effects (*k*_1_, *k*_2_ and *K*) were large (Table 2).

Sensitivity of model predictions—viral load and effector response—pertaining to the progressive disease and viremic control stable steady states, separately, to input parameter values, was tested by the calculation of partial rank correlation coefficients [80] (S6 Fig). The predictions were most sensitive to viral production and clearance rates and parameters associated with CTL stimulation.

We also obtained steady state solutions by setting the left-hand sides of all Eqs (13)–(20) to zero and solving the resulting coupled non-linear equations in Mathematica with the obtained parameter values. We subsequently performed linear stability analysis, by computing the eigenvalues of the Jacobian matrix of the above dynamical system, to assess the stability of the steady states (parameter bifurcation plots in Fig 5 and S15 Fig).

Nishimura *et al*. [10] administered anti-CD8*α* antibodies to 6 macaques (MVJ, DEMR, DEWL, DEWP, MAF and DFIK) that resulted in depletion of CD8^+^ T, NK, NKT, and *γδ* T cells. All macaques exhibited a sharp increase in viremia, followed by reassertion of control in 5 macaques, and loss of control in one macaque (DFIK). Nishimura *et al*. [10] also administered anti-CD8*β* antibodies to 3 macaques (MVJ, DEMR and DEWL) that only depleted CD8^+^ T cells, resulting in a smaller increase in viremia compared to the administration of anti-CD8*α* antibodies. A summary of the treatments administered [10] is in S10 Table. We captured these observations with our model.

We assumed that the depleting effects of anti-CD8*α* and anti-CD8*β* antibodies started at time points *θ*_*α*_ and *θ*_*β*_, respectively, at which points we reduced the effector populations by fractions *ζ*_*α*_ and *ζ*_*β*_. Further, we assumed that anti-CD8*α* antibodies neutralized all host effector functions, which we modeled by setting *m* = 0 for a duration *θ*_*m*_ representing the residence time of the depleting antibodies. We fit *θ*_*α*_, *θ*_*β*_, *θ*_*m*_, *ζ*_*α*_, and *ζ*_*β*_ to viral load data following the administration of depleting antibodies from all macaques (best-fits in phase IV of Fig 2; summarized in S10 Table). Because explicit dynamics of the anti-CD8 antibodies were not available from Nishimura *et al*. [10], fitting all the available data including following CD8 depletion with Monolix consistently yielded poor fits, especially in phase II (green region in Fig 2), and often with unrealistic parameter estimates. Therefore, we first fit the data from all 10 controller macaques treated with bNAbs and obtained macaque-specific parameter estimates from the population fits excluding data post CD8 depletion. Next, with these parameters fixed, we fit viral loads upon CD8 depletion to individual macaque data for the 6 macaques that underwent CD8 depletion (phase IV in Fig 2). We thus obtained estimates of parameters corresponding to both CD8*α* and CD8*β* depletion in 3 macaques (MVJ, DEMR and DEWL) and parameters corresponding to CD8*α* depletion in the other 3 macaques (DEWP, MAF and DFIK). In the former three, viral loads in the phase III between depletions were not fit because they were undetectable. Fits were obtained using the ‘multistart’ and ‘lsqcurvefit’ tools along with the ode15s solver in Matlab (in.mathworks.com).

### Model building strategy

Here, we discuss the way in which we developed our model (Eqs 1–8) beginning with the basic model of viral dynamics. We first checked whether the basic model, together with the dynamics of the latent reservoir but without an explicit effector response, as suggested previously to describe viral blips during ART [81], could capture the viral load data. Administered Abs were assumed to increase the viral clearance rate in a dose-dependent manner. The scaled model equations were:

$$\begin{array}{ccc} T^{*} & = & T\frac{d_{T}}{\lambda_{T}},\;\;I^{*}=I\frac{d_{T}}{\lambda_{T}},\;\;L^{*}=L\frac{d_{T}}{\lambda_{T}},\;\;p^{*}=p\frac{\lambda_{T}}{d_{T}} \end{array}$$ (24)

$$\begin{array}{ccc} \frac{dT^{*}}{dt} & = & d_{T}(1-T^{*})-\beta VT^{*} \end{array}$$ (25)

$$\begin{array}{ccc} \frac{dI^{*}}{dt} & = & (1-f_{L})\beta VT^{*}+aL^{*}-d_{I}I^{*} \end{array}$$ (26)

$$\begin{array}{ccc} \frac{dL^{*}}{dt} & = & f_{L}\beta VT^{*}+(\psi -a-d_{L})L^{*} \end{array}$$ (27)

$$\begin{array}{ccc} \frac{dV}{dt} & = & p^{*}I^{*}-(c+A)V \end{array}$$ (28)

where bNAb pharmacodynamics (*A*_1_ and *A*_2_) remained as before (Eqs 19 and 20). In this model, a fraction (*f*_*L*_ = 10^−6^) of uninfected CD4^+^ infections yield latently-infected cells [82], *L*, and the rest, productively-infected cells, *I*. Cells *L* proliferate, get activated, and die at the per capita rates *ψ*, *a*, and *d*_*L*_ = 0.004 day^-1^ (fixed based on Ref. [18]), respectively. The model could not capture the *in vivo* temporal viremic patterns (S9 Fig and S4 Table), reaffirming the experimentally observed [10] role of the effector response in establishing robust viremic control. Therefore, we next incorporated the effecter response following a model of post-ART control [18]. The model considered both activation and exhaustion of CD8 T cells. The scaled model equations were:

$$\begin{array}{ccc} T^{*} & = & T\frac{d_{T}}{\lambda_{T}},\;\;I^{*}=I\frac{d_{T}}{\lambda_{T}},\;\;L^{*}=L\frac{d_{T}}{\lambda_{T}},\;\;E^{*}=E\frac{d_{E}}{\rho } \end{array}$$ (29)

$$\begin{array}{ccc} p^{*} & = & p\frac{\lambda_{T}}{d_{T}},\;\;m^{*}=m\frac{\rho }{d_{E}},\;\;f^{*}=f\frac{d_{T}}{\lambda_{T}},\;\;\phi_{1}^{*}=\phi_{1}\frac{d_{T}}{\lambda_{T}},\;\;\phi_{2}^{*}=\phi_{2}\frac{d_{T}}{\lambda_{T}} \end{array}$$ (30)

$$\begin{array}{ccc} \frac{dT^{*}}{dt} & = & d_{T}(1-T^{*})-\beta VT^{*} \end{array}$$ (31)

$$\begin{array}{ccc} \frac{dI^{*}}{dt} & = & (1-f_{L})\beta VT^{*}+aL^{*}-(d_{I}+m^{*}E^{*})I^{*} \end{array}$$ (32)

$$\begin{array}{ccc} \frac{dL^{*}}{dt} & = & f_{L}\beta VT^{*}+(\psi -a-d_{L})L^{*} \end{array}$$ (33)

$$\begin{array}{ccc} \frac{dV}{dt} & = & p^{*}I^{*}-(c+A)V \end{array}$$ (34)

$$\begin{array}{ccc} \frac{dE^{*}}{dt} & = & d_{E}+\frac{k_{E}E^{*}(I^{*}+f^{*}AV)}{\left(\phi_{1}^{*}+I^{*}+f^{*}AV\right)}-(\frac{\xi I^{*}}{\phi_{2}^{*}+I^{*}}+d_{E})E^{*} \end{array}$$ (35)

Here, we recognized additionally that activation of effector cells could be facilitated not only by infected cells but also by bNAb-opsonized virions leading to enhanced antigen uptake and presentation by antigen presenting cells. We thus modified the activation term by adding the contribution from bNAbs (Eq 35). Again, bNAb pharmacodynamics (*A*_1_ and *A*_2_) remained the same as Eqs 19 and 20. *ϕ*_1_ and *ϕ*_2_ were the effector activation and exhaustion thresholds, respectively. We followed the population-based fitting strategy above. Latent pool parameters were fixed [18]: *ψ* = 0.0045 day^-1^, *a* = 0.001 day^-1^, and *d*_*L*_ = 0.004 day^-1^. The model failed to capture the viral load patterns observed within the 200 stochastic realizations employed (S10 Fig and S5 Table).

One possibility behind the failure to fit data with the model above was the approximate characterization of CTL exhaustion. The model assumed that exhaustion depended on the magnitude of the instantaneous antigen exposure and not on sustained or cumulative stimulation, whereas experiments suggest the latter [72]. Exhaustion arising from cumulative antigen stimulation has been described previously [19]. We therefore employed the latter formalism for exhaustion next. The resulting equations yielded the main model (Eqs 1–8).

A crucial parameter in this model is the Hill coefficient for exhaustion, *n*, which is unknown. We initially fixed *n* = 3 according to the original model [19], but, as outlined above, failed to capture the data. Specifically, viral load patterns upon effector depletion were not captured (S11 Fig and S6 Table). Upon effector depletion with anti-CD8*α* and anti-CD8*β* antibodies for the macaques DEWP and MVJ, respectively, while measurements by Nishimura *et al*., [10] showed re-establishment of control, fits exhibited loss of control and attainment of the high viremic steady state. Therefore, we attempted fitting with both a sharper (*n* = 4) and a more gradual (*n* = 1) exhaustion switch, the latter similar to the post-ART control model [18]. While the model with *n* = 1 failed to capture the viral loads (S12 Fig and S7 Table), the model with *n* = 4 (the main model) quantitatively captured all the *in vivo* data (Figs 2–4 and S1 Table).

Finally, to build a parsimonious model that quantitatively captured the mechanism of long-term viremic control, we tested variants of our model without specific bNAb mediated effects such as enhanced antigen clearance (no *AV* term in Eq 15) or without enhanced antigen uptake and subsequent effector elicitation (no f*AV term in Eq 16). The model without antigen clearance failed to capture the viral load patterns (S13 Fig) while the model without enhanced effector elicitation yielded poorer fits (S14 Fig, with higher AIC (Table 3). This reaffirmed the importance of pleiotropic effects of bNAbs underlying the orchestration of viremic control.

## Supporting information

S1 Fig Estimation of parameter population means. Maximum likelihood estimation of the population parameter means (*μ*; Methods) with the stochastic approximation expectation-maximization (SAEM) algorithm implemented in Monolix for non-linear mixed effects modeling. Evolution of the parameter population mean values over the iterations of the algorithm are displayed. The vertical dashed line indicates the transition from the ‘exploratory’ phase, where extensive parameter sampling occurs to obtain the approximate location of the maximum likelihood, to the ‘smoothing’ phase, where accurate convergence to the maximum likelihood occurs.
(TIF)

S2 Fig Estimation of the standard deviations of random effects. The same as S1 Fig but for the standard deviations of the parameter random effect distributions (*σ*; Methods).
(TIF)

S3 Fig Estimation of the parameters of the residual error model. The same as S1 Fig but for the parameters of the combined residual error model (*a* and *b* for *A*_1_, *A*_2_ and *V*; see Eq 23 in Methods). (*b*_*Virus*_ ∼ 0, indicating that a constant error model would have sufficed for estimating *V*). The overall convergence indicator for S1–S3 Figs is shown in magenta and indicates that the SAEM algorithm has converged and maximum likelihood achieved.
(TIF)

S4 Fig AIC estimation and convergence. Convergence of the estimated Akaike information criteria, obtained by empirical log-likelihood estimation from an importance sampling Monte Carlo method implemented in Monolix.
(TIF)

S5 Fig Individual parameter distributions. Empirical distributions (blue histograms; probability densities) of the individual parameters simulated from the estimated conditional distributions using Markov chain Monte Carlo methods (implemented in Monolix). The overlaid theoretical parameter distributions (red lines) are defined by their respective statistical models (Methods, Eqs 21 and 22) along with the estimated population mean and random effects.
(TIF)

S6 Fig Parameter sensitivity. Sensitivity of the predicted viral load and effector response steady states, pertaining to viremic control (blue) and progressive disease (red), to the model input parameters, estimated using partial rank correlation coefficients with sample size = 100000 (Methods). PRCC values indicate sensitivity of the sum of square errors (SSE) between model predictions of viral load and effector response with baseline parameter values, to those upon varying the parameters. The range of variation for the input parameters was based on the actual parameter distributions obtained from the fits (S5 Fig). Fixed parameters such as the viral clearance rate *c* were uniformly varied in a range between half to twice of the fixed value. *Δ* is a dummy control variable that is not part of the model and hence sets the threshold for significance.
(TIF)

S7 Fig Influence of the latent reservoir. Fits to *in vivo* data of macaque MVJ (red—untreated, blue—early bNAb therapy) and dynamics of the effector response and level of exhaustion, both with (dashed lines) and without (solid lines) the latent reservoir in the main model. When the latent reservoir is included, productively and latently infected cellular dynamics (including the latent pool parameters) are based on the model of post-ART control (Methods, Eqs 32 and 33).
(TIF)

S8 Fig Main model with varying effector proliferation (*k*_*E*_). Fitting with the main model but with varying effector proliferation (*k*_*E*_ in Eq 16) following the procedure outlined in Methods yielded good fits (blue) to the data (parameters in S3 Table) but with a higher AIC (Table 3). Corresponding predictions without treatment are in red.
(TIF)

S9 Fig Basic viral dynamics model without an effector response. Fitting with a basic viral dynamics model without a effector response (Eqs 25–28) following the procedure outlined in Methods yielded poor fits (blue) to the data (parameters in S4 Table). Corresponding predictions without treatment are in red.
(TIF)

S10 Fig Model based on post-ART control. Fitting with the post-ART control model (Eqs 31–35) following the procedure outlined in the Methods yielded poor fits (blue) to the data (parameters in S5 Table). Corresponding predictions without treatment are in red.
(TIF)

S11 Fig Hill coefficient, *n* = 3. Fitting our model (Eqs 13–20) with a Hill coefficient *n* = 3 following the procedure outlined in Methods yielded poor fits (blue) to the data after effector depletion (parameters in S6 Table; see comments in Table 3). Corresponding predictions without treatment are in red.
(TIF)

S12 Fig Hill coefficient, *n* = 1. Fitting our model (Eqs 13–20) with a Hill coefficient *n* = 1 following the procedure outlined in Methods yielded poor fits (blue) to the data (parameters in S7 Table). Corresponding predictions without treatment are in red.
(TIF)

S13 Fig Fits of model without enhanced antigen clearance by bNAbs. Fitting our model (Eqs 13–20) without enhanced antigen clearance by bNAbs (no *AV* term in Eq 15) following the procedure outlined in the Methods yielded poor fits (blue) to the data (parameters in S8 Table). Corresponding predictions without treatment are in red.
(TIF)

S14 Fig Fits of model without enhanced effector elicitation by bNAbs. Fitting our model (Eqs 13–20) without enhanced antigen uptake and subsequent effector elicitation by bNAbs (no f*AV term in Eq 16) following the procedure outlined in the Methods yielded poorer fits (blue) to the data (parameters in S9 Table) compared to the main model (Fig 2) and yielded a higher AIC (Table 3). Corresponding model predictions without treatment are in red.
(TIF)

S15 Fig Bistability and bifurcation diagrams. Steady states of our model (Methods) obtained by varying underlying parameters (different panels) one at a time over wide ranges about their values listed in Tables 1 and 2. The stable states of high and low viremia are shown in red and blue, respectively. Black lines represent unstable steady states. Parameters: *β*—infectivity of virions, *c*—viral clearance rate, *d*_*E*_—death rate of effectors, *d*_*I*_—death rate of infected CD4^+^ T cells, *k*_*E*_—proliferation rate of effectors, m*—rate at which effectors kill infected cells, ϕ*—threshold for effector activation as well as level of exhaustion, p*—burst size, and *ξ*—maximal rate of effector exhaustion.
(TIF)

S1 Table Individual parameter estimates for treated macaques obtained by simultaneously fitting our model (Eqs 13–20) to *V*, *A*_1_ and *A*_2_ across both untreated macaques and responders (Methods, Figs 2–4). Parameters pertaining to effector depletion experiments with anti-CD8*α* and anti-CD8*β* antibodies were obtained from individual fits (best fits in Fig 2). The units of all the parameters are the same as in Table 2; *ζ*_*α*_ and *ζ*_*β*_ are dimensionless while *θ*_*m*_, *θ*_*α*_, *θ*_*β*_ are in days. (NA—not applicable; see S10 Table).
(PDF)

S2 Table Individual parameter estimates for the ten untreated macaques obtained by simultaneously fitting our model (Eqs 13–20) to *V*, *A*_1_ and *A*_2_ across both untreated macaques and responders (Methods and Fig 4). (PDF)

S3 Table Individual parameter estimates obtained as in S1 Table but with varying effector proliferation rate, *k*_*E*_ (Methods and S8 Fig for details). (PDF)

S4 Table Individual parameter estimates obtained by fitting a basic viral dynamics model without an explicit effector response (Eqs 25–28; Methods and S9 Fig for details). Here, *d*_*L*_ was fixed at 0.004 day^-1^.
(PDF)

S5 Table Individual parameter estimates obtained by fitting the post-ART control model (Eqs 31–35; Methods and S10 Fig for details). (PDF)

S6 Table Individual parameter estimates as obtained in S1 Table but with the Hill coefficient *n* = 3 (Methods and S11 Fig for details). (PDF)

S7 Table Individual parameter estimates as obtained in S1 Table but with the Hill coefficient *n* = 1 (Methods and S12 Fig for details). (PDF)

S8 Table Individual parameter estimates for treated macaques obtained by simultaneously fitting models without enhanced antigen clearance by bNAbs (no *AV* term in Eq 15) to *V*, *A*_1_ and *A*_2_ across both untreated macaques and responders (Methods and S13 Fig for details). (PDF)

S9 Table Individual parameter estimates for treated macaques obtained by simultaneously fitting models without enhanced antigen uptake and subsequent effector elicitation by bNAbs (no f*AV term in Eq 16) to *V*, *A*_1_ and *A*_2_ across both untreated macaques and responders (Methods and S14 Fig for details). (PDF)

S10 Table Summary table of macaques from Nishimura *et al*. which were subjected to early bNAb therapy, with Macaque ID, initial viral inoculum, route of administration, whether anti-CD8*α* Ab- and anti-CD8*β* Ab-mediated effector depletion was performed, and whether these macaques regained viremic control subsequently. Macaques to which only anti-CD8*α* Abs, or both anti-CD8*α* and anti-CD8*β* Abs were administered, have their IDs colored blue and purple, respectively. Non-controller macaque IDs are colored orange and their data was not considered for fitting.
(PDF)

S1 Data Supplementary excel file containing digitized viral loads and serum concentrations of bNAbs 3BNC117 and 10-1074 with time, for all bNAb-treated and untreated macaques from Nishimura *et al*. [10]. (XLSX)
