# Supplementary figures and images for: Early exposure to broadly neutralizing antibodies may trigger a dynamical switch from progressive disease to lasting control of SHIV infection
Source: PLoS Comput Biol. 2020 Aug 20;16(8):e1008064. doi: 10.1371/journal.pcbi.1008064 (PMC7462315; doi:10.1371/journal.pcbi.1008064)

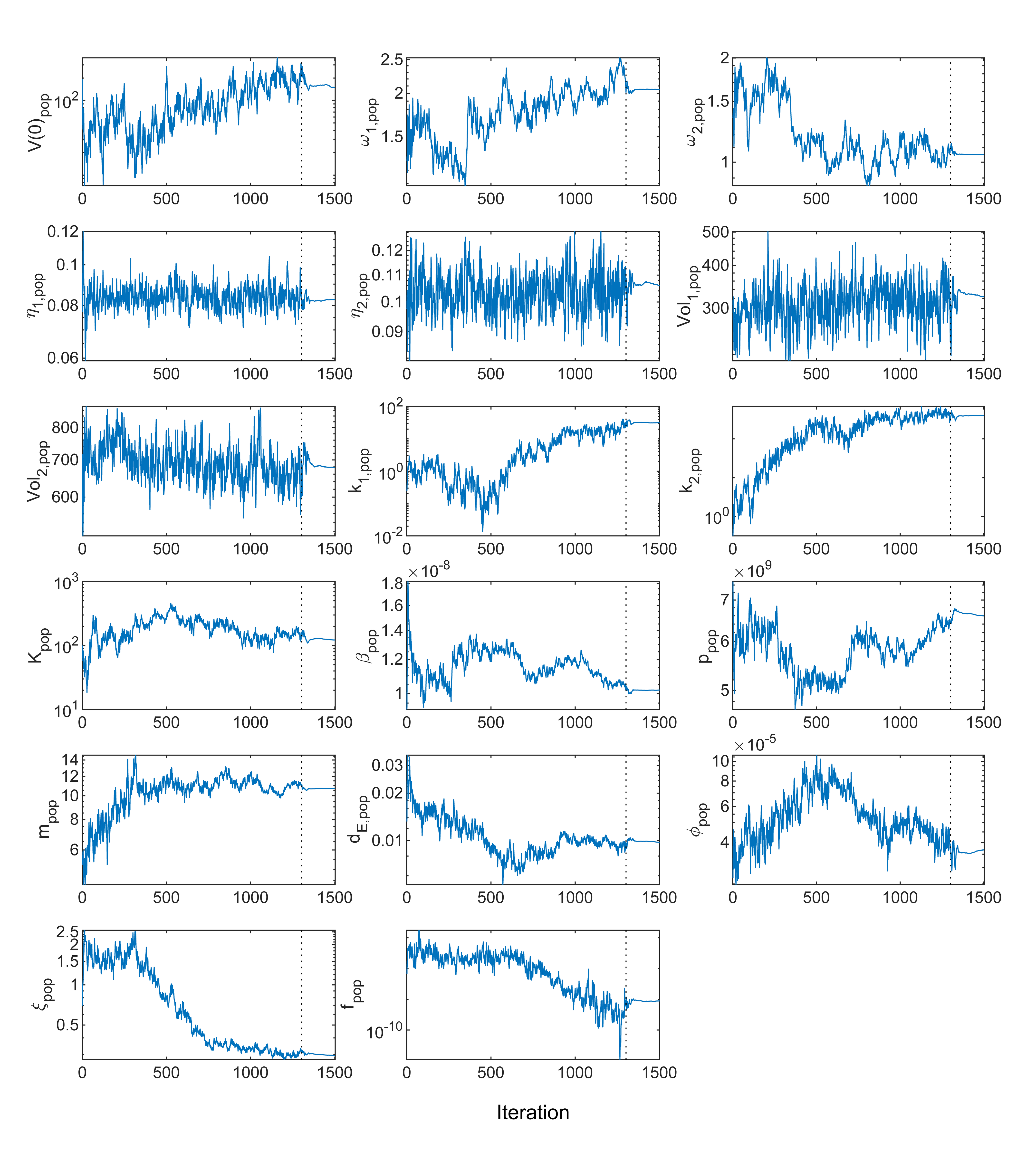

Supplement: S1 Fig — Maximum likelihood estimation of the population parameter means (μ; Methods) with the stochastic approximation expectation-maximization (SAEM) algorithm implemented in Monolix for non-linear mixed effects modeling. Evolution of the parameter population mean values over the iterations of the algorithm are displayed. The vertical dashed line indicates the transition from the ‘exploratory’ phase, where extensive parameter sampling occurs to obtain the approximate location of the maximum likelihood, to the ‘smoothing’ phase, where accurate convergence to the maximum likelihood occurs. (TIF) [file pcbi.1008064.s001.tif]

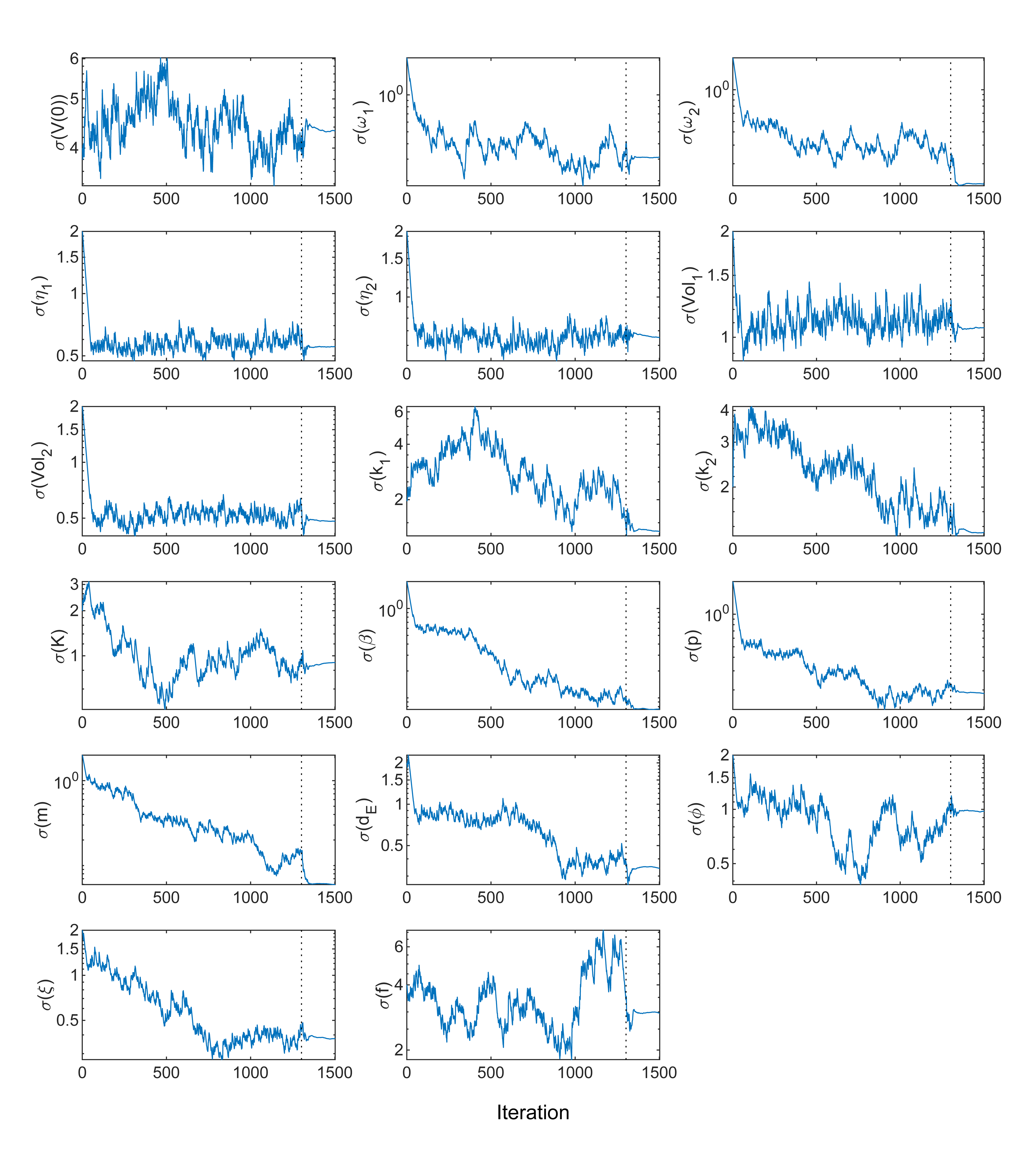

Supplement: S2 Fig — The same as S1 Fig but for the standard deviations of the parameter random effect distributions (σ; Methods). (TIF) [file pcbi.1008064.s002.tif]

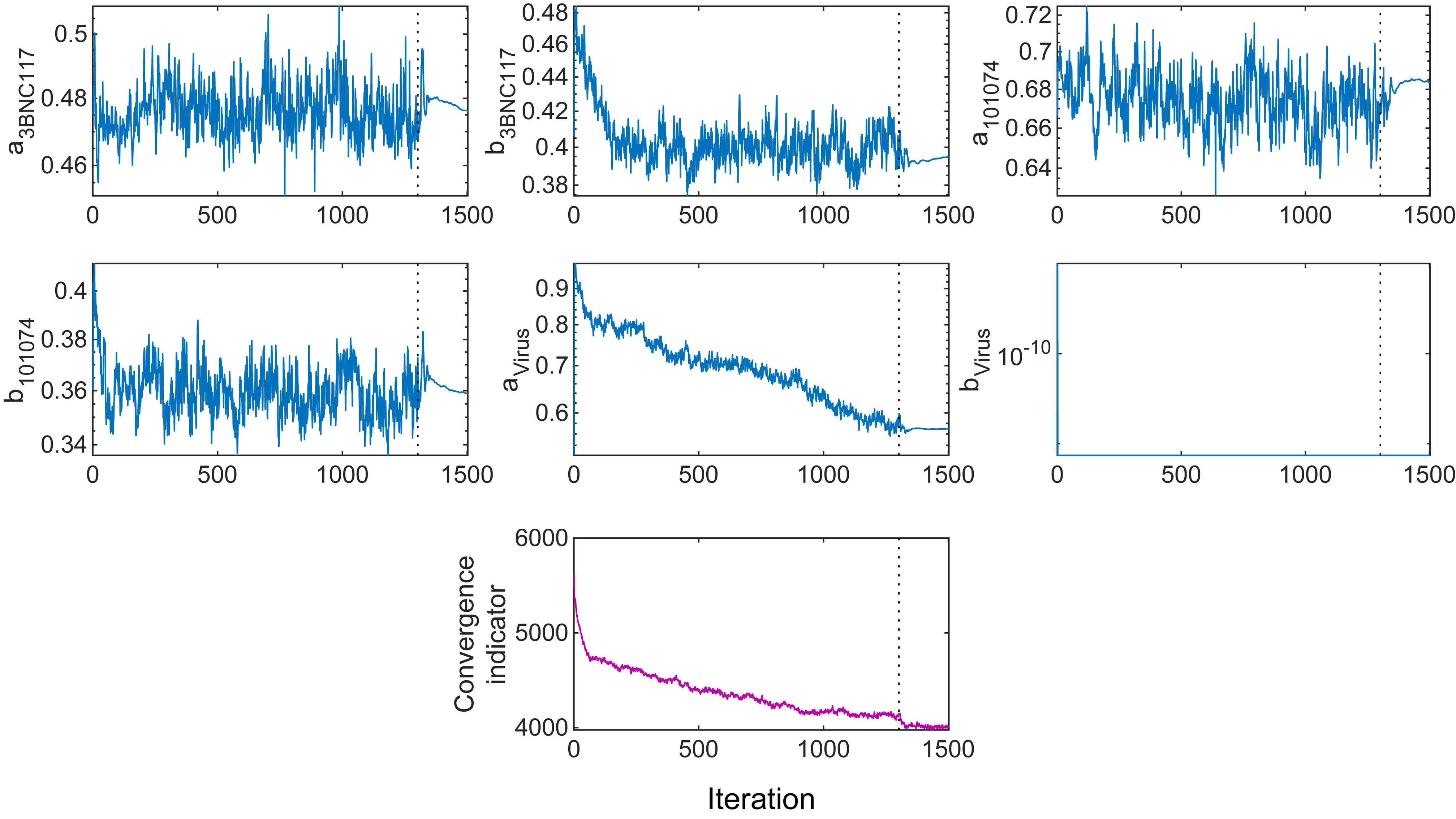

Supplement: S3 Fig — The same as S1 Fig but for the parameters of the combined residual error model (a and b for A1, A2 and V; see Eq 23 in Methods). (bVirus ∼ 0, indicating that a constant error model would have sufficed for estimating V). The overall convergence indicator for S1–S3 Figs is shown in magenta and indicates that the SAEM algorithm has converged and maximum likelihood achieved. (TIF) [file pcbi.1008064.s003.tif]

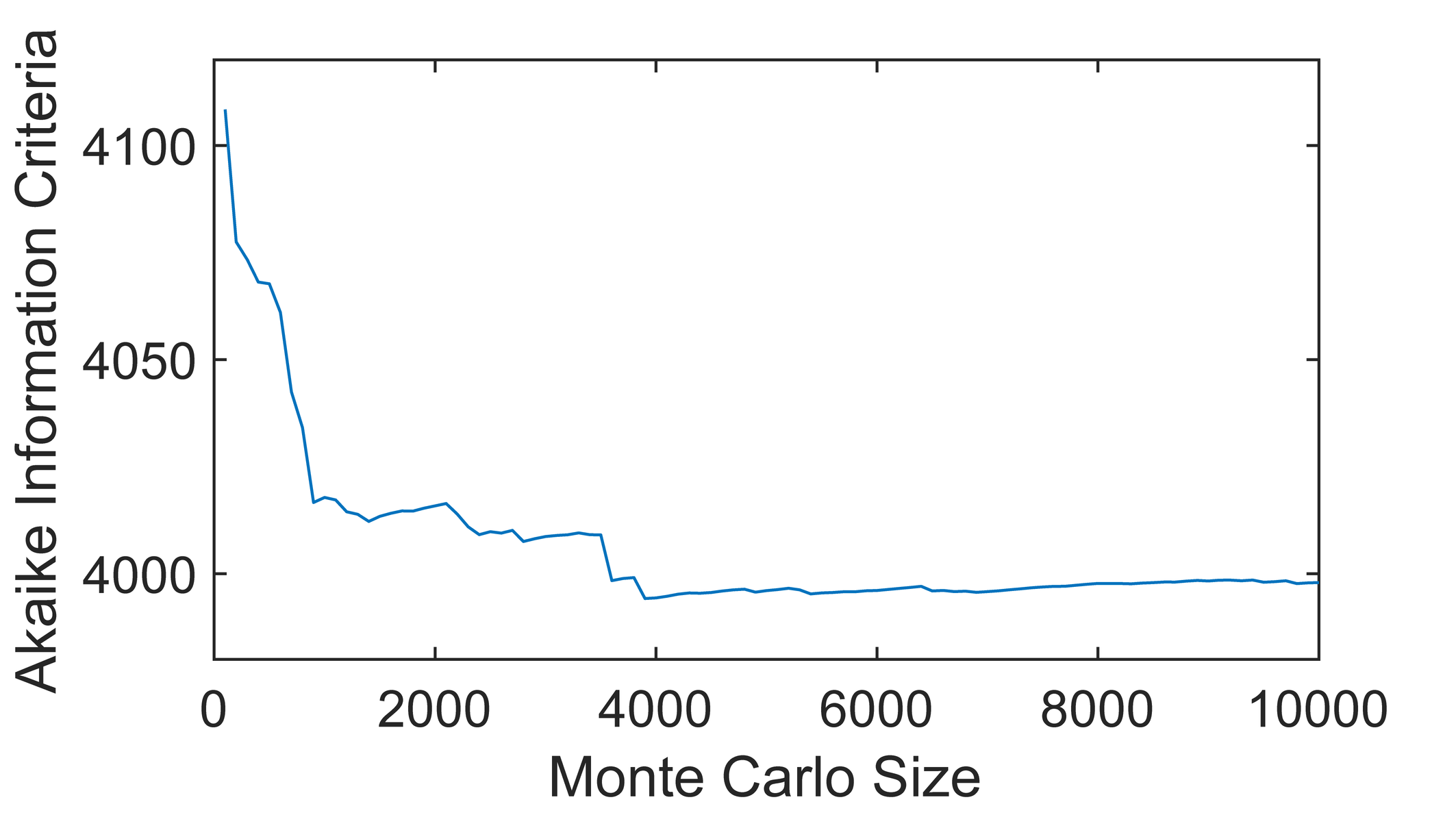

Supplement: S4 Fig — Convergence of the estimated Akaike information criteria, obtained by empirical log-likelihood estimation from an importance sampling Monte Carlo method implemented in Monolix. (TIF) [file pcbi.1008064.s004.tif]

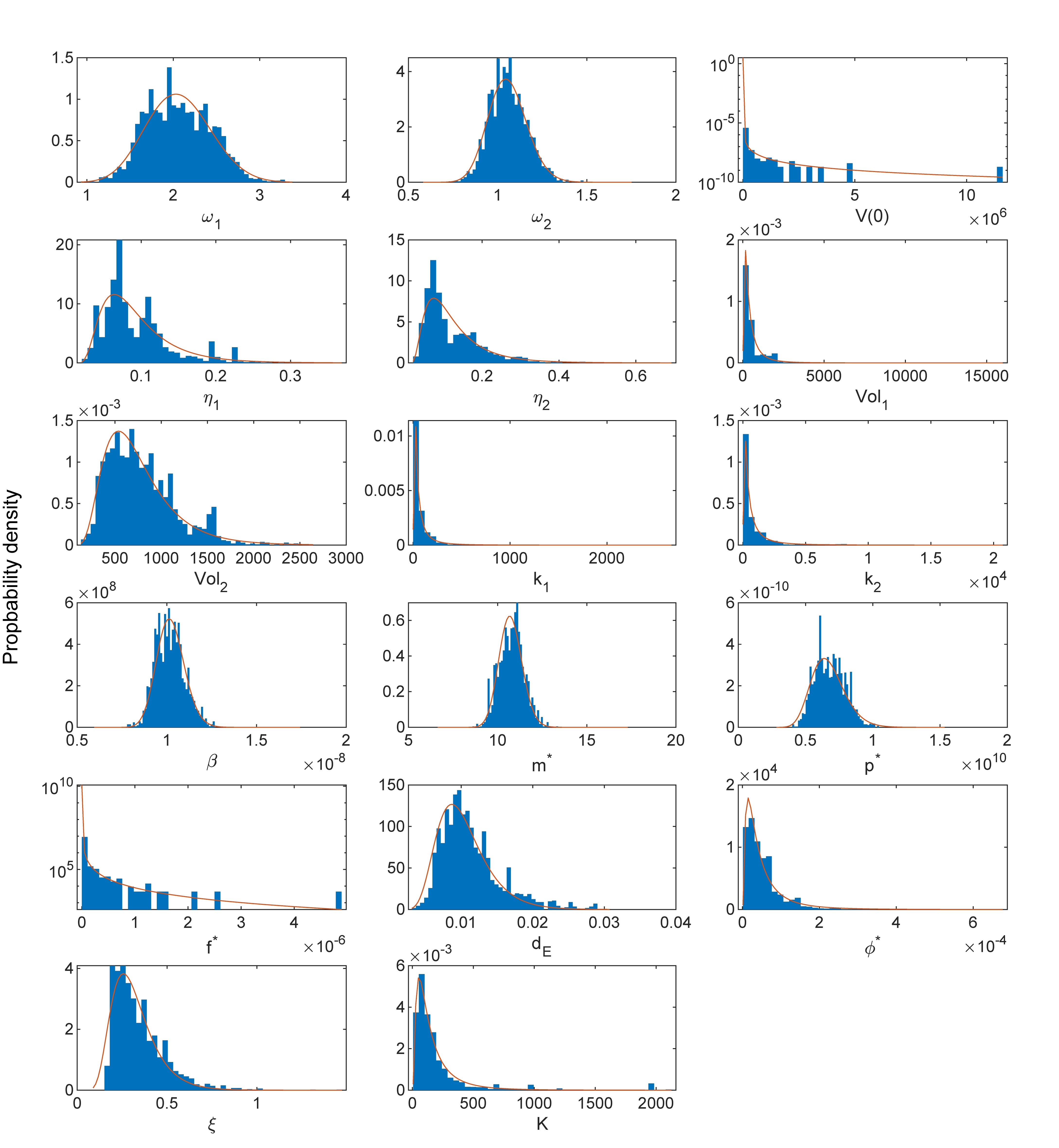

Supplement: S5 Fig — Empirical distributions (blue histograms; probability densities) of the individual parameters simulated from the estimated conditional distributions using Markov chain Monte Carlo methods (implemented in Monolix). The overlaid theoretical parameter distributions (red lines) are defined by their respective statistical models (Methods, Eqs 21 and 22) along with the estimated population mean and random effects. (TIF) [file pcbi.1008064.s005.tif]

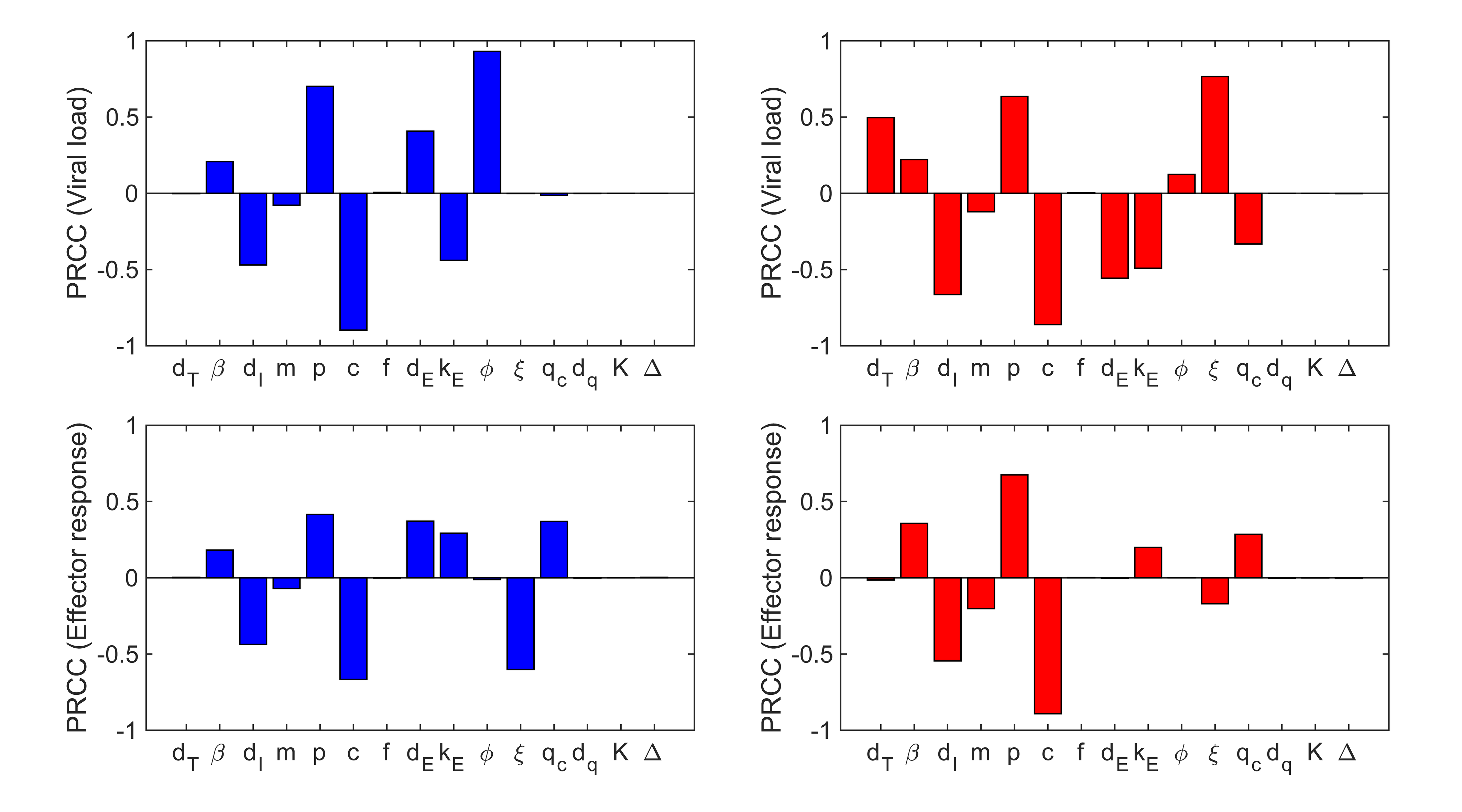

Supplement: S6 Fig — Sensitivity of the predicted viral load and effector response steady states, pertaining to viremic control (blue) and progressive disease (red), to the model input parameters, estimated using partial rank correlation coefficients with sample size = 100000 (Methods). PRCC values indicate sensitivity of the sum of square errors (SSE) between model predictions of viral load and effector response with baseline parameter values, to those upon varying the parameters. The range of variation for the input parameters was based on the actual parameter distributions obtained from the fits (S5 Fig). Fixed parameters such as the viral clearance rate c were uniformly varied in a range between half to twice of the fixed value. Δ is a dummy control variable that is not part of the model and hence sets the threshold for significance. (TIF) [file pcbi.1008064.s006.tif]

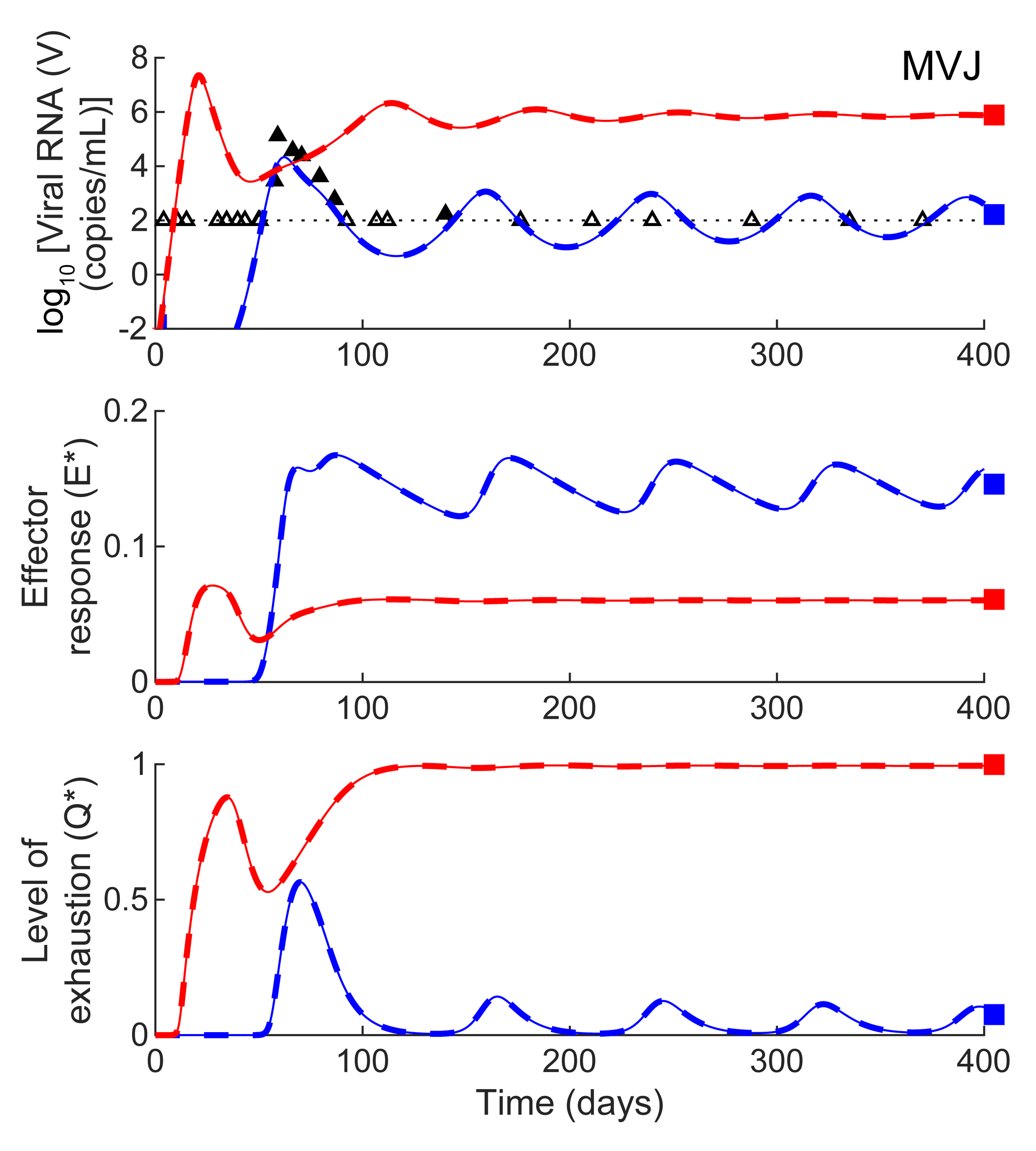

Supplement: S7 Fig — Fits to in vivo data of macaque MVJ (red—untreated, blue—early bNAb therapy) and dynamics of the effector response and level of exhaustion, both with (dashed lines) and without (solid lines) the latent reservoir in the main model. When the latent reservoir is included, productively and latently infected cellular dynamics (including the latent pool parameters) are based on the model of post-ART control (Methods, Eqs 32 and 33). (TIF) [file pcbi.1008064.s007.tif]

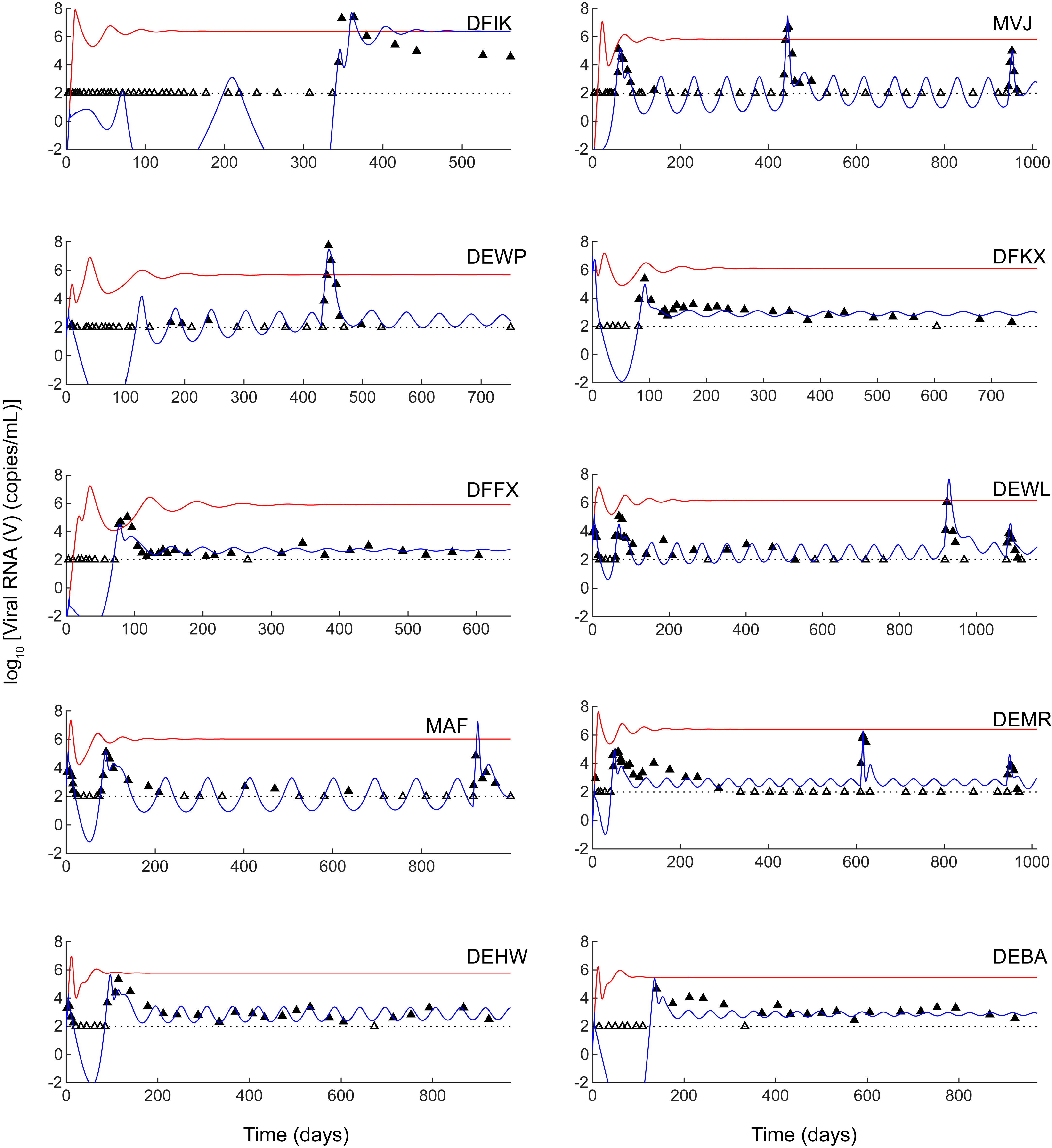

Supplement: S8 Fig — Fitting with the main model but with varying effector proliferation (kE in Eq 16) following the procedure outlined in Methods yielded good fits (blue) to the data (parameters in S3 Table) but with a higher AIC (Table 3). Corresponding predictions without treatment are in red. (TIF) [file pcbi.1008064.s008.tif]

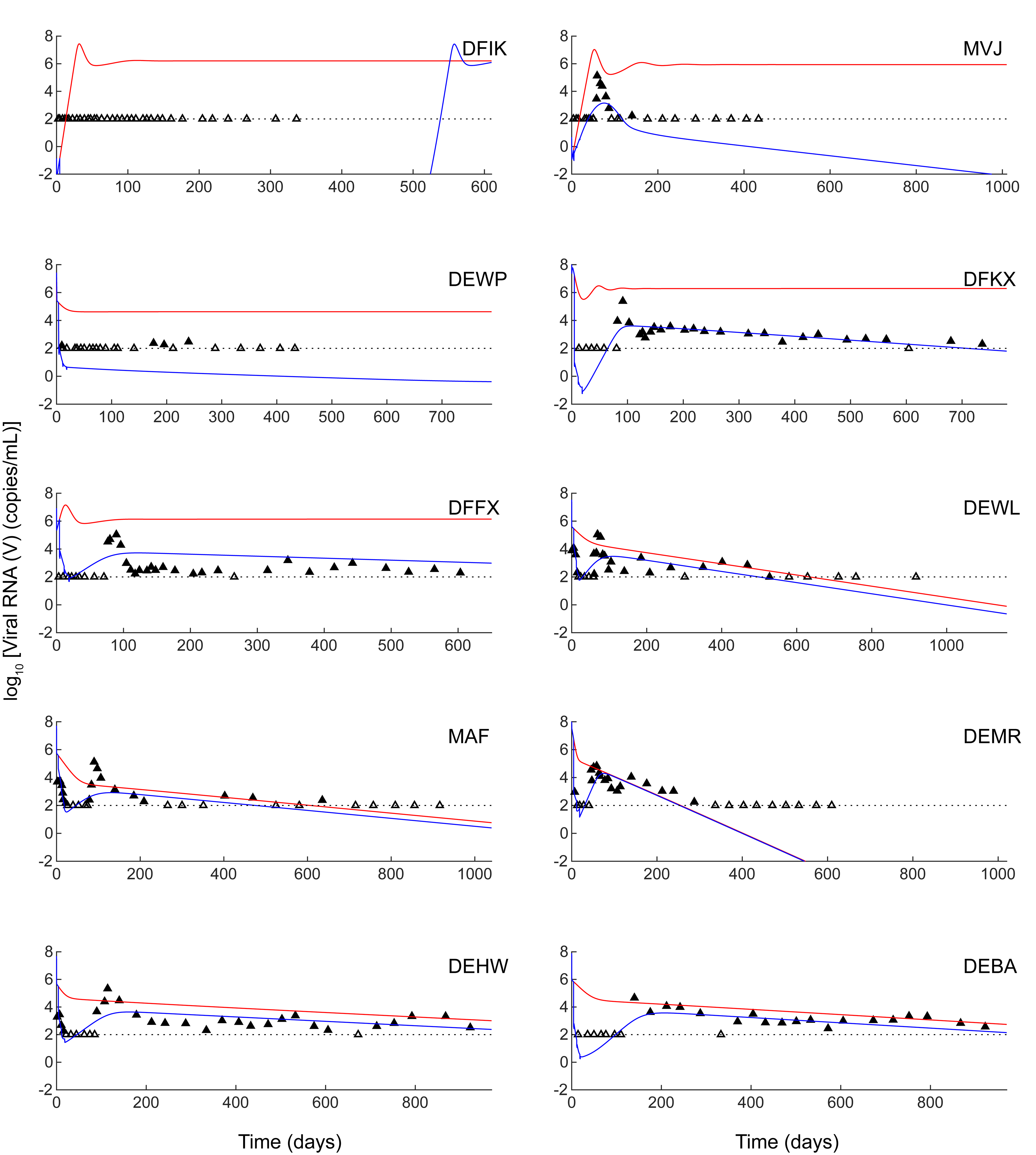

Supplement: S9 Fig — Fitting with a basic viral dynamics model without a effector response (Eqs 25–28) following the procedure outlined in Methods yielded poor fits (blue) to the data (parameters in S4 Table). Corresponding predictions without treatment are in red. (TIF) [file pcbi.1008064.s009.tif]

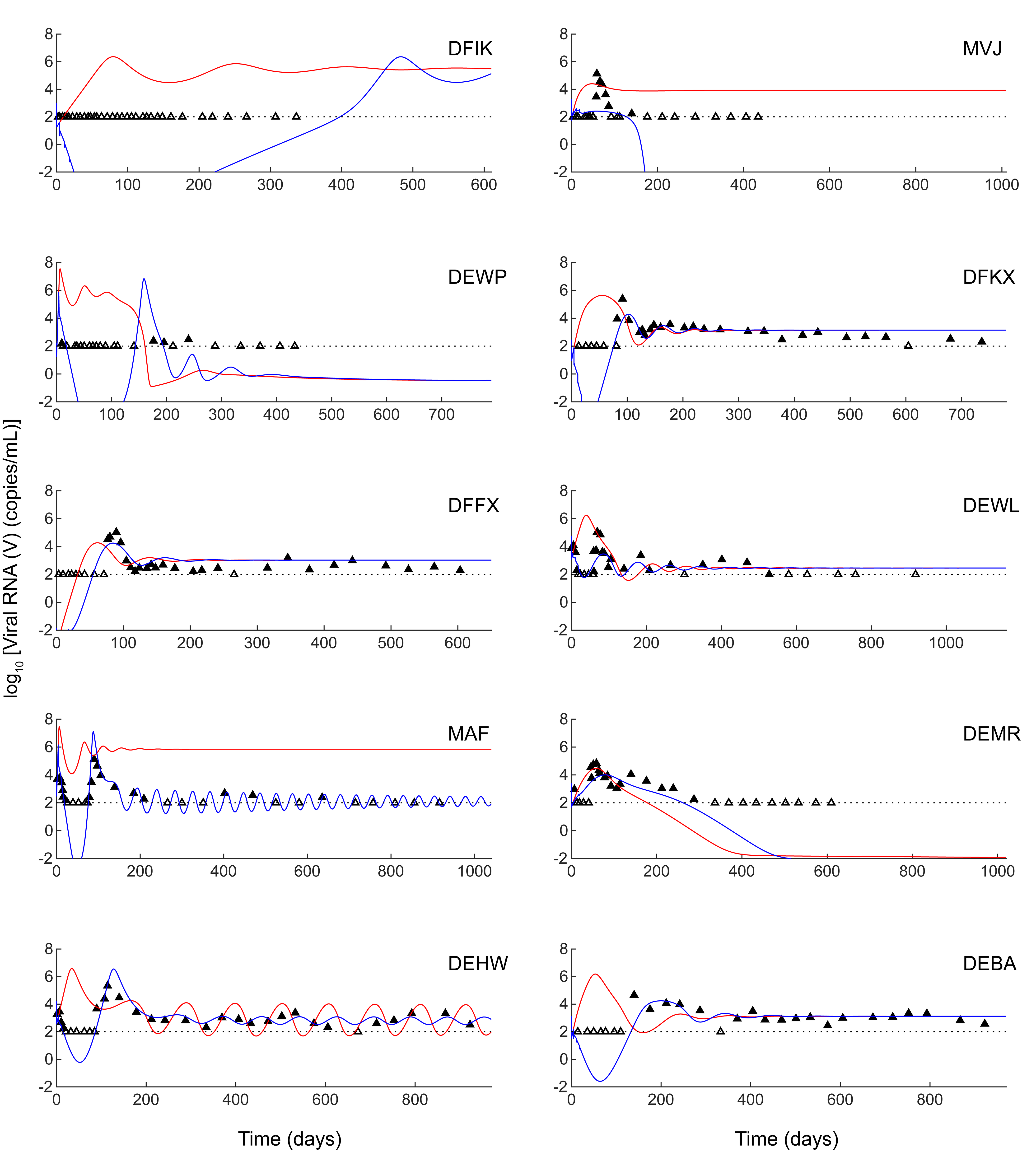

Supplement: S10 Fig — Fitting with the post-ART control model (Eqs 31–35) following the procedure outlined in the Methods yielded poor fits (blue) to the data (parameters in S5 Table). Corresponding predictions without treatment are in red. (TIF) [file pcbi.1008064.s010.tif]

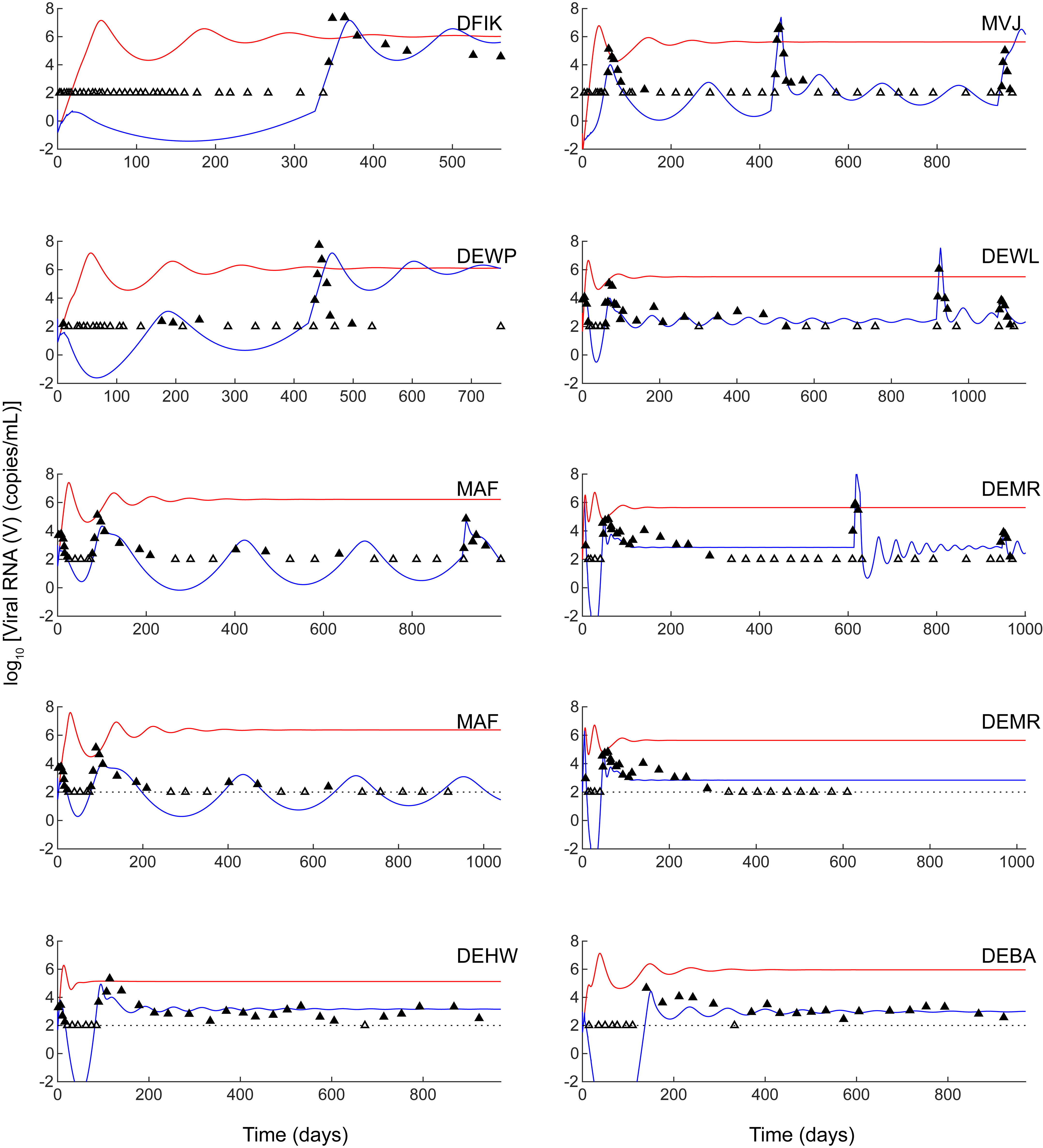

Supplement: S11 Fig — Fitting our model (Eqs 13–20) with a Hill coefficient n = 3 following the procedure outlined in Methods yielded poor fits (blue) to the data after effector depletion (parameters in S6 Table; see comments in Table 3). Corresponding predictions without treatment are in red. (TIF) [file pcbi.1008064.s011.tif]

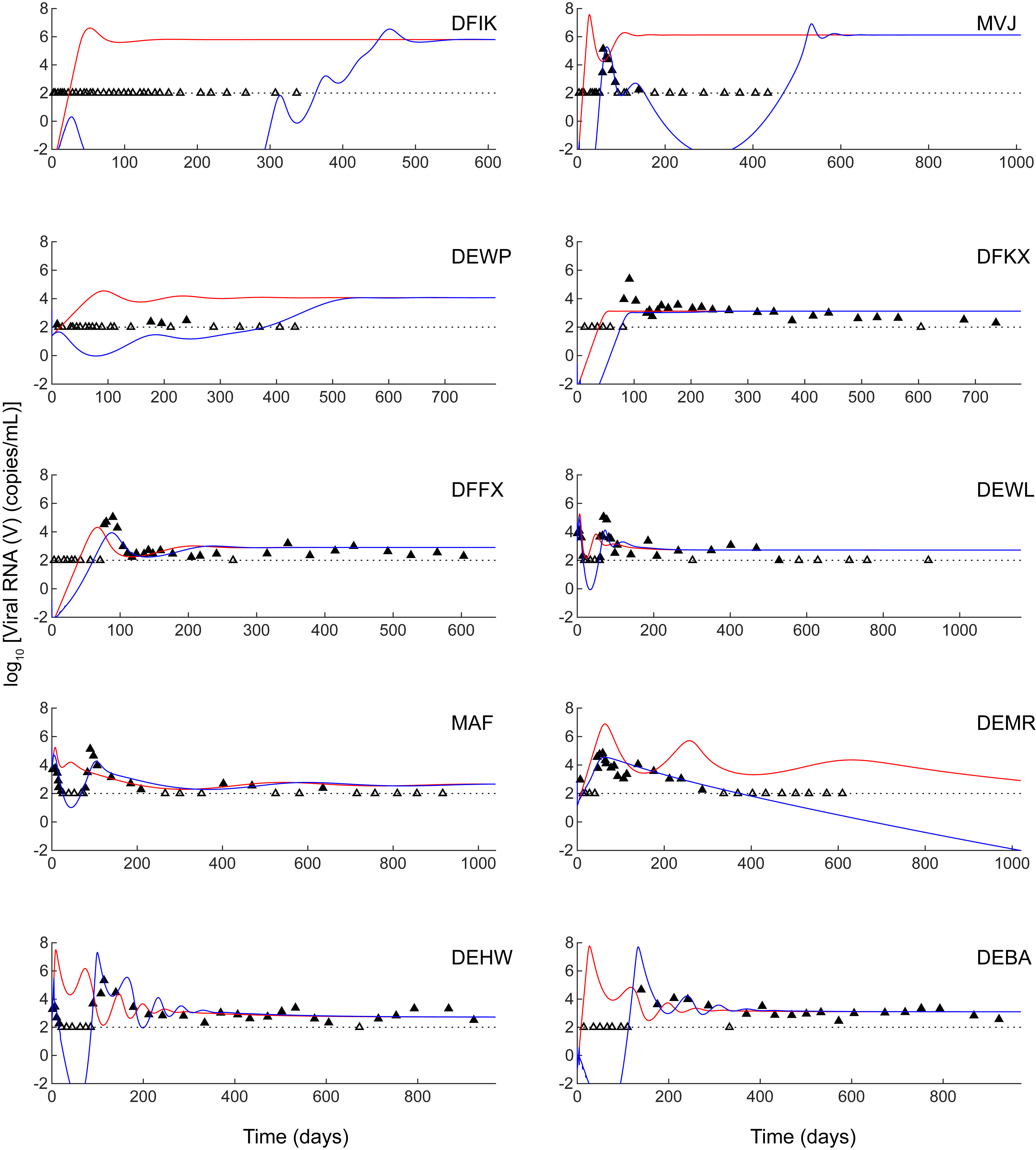

Supplement: S12 Fig — Fitting our model (Eqs 13–20) with a Hill coefficient n = 1 following the procedure outlined in Methods yielded poor fits (blue) to the data (parameters in S7 Table). Corresponding predictions without treatment are in red. (TIF) [file pcbi.1008064.s012.tif]

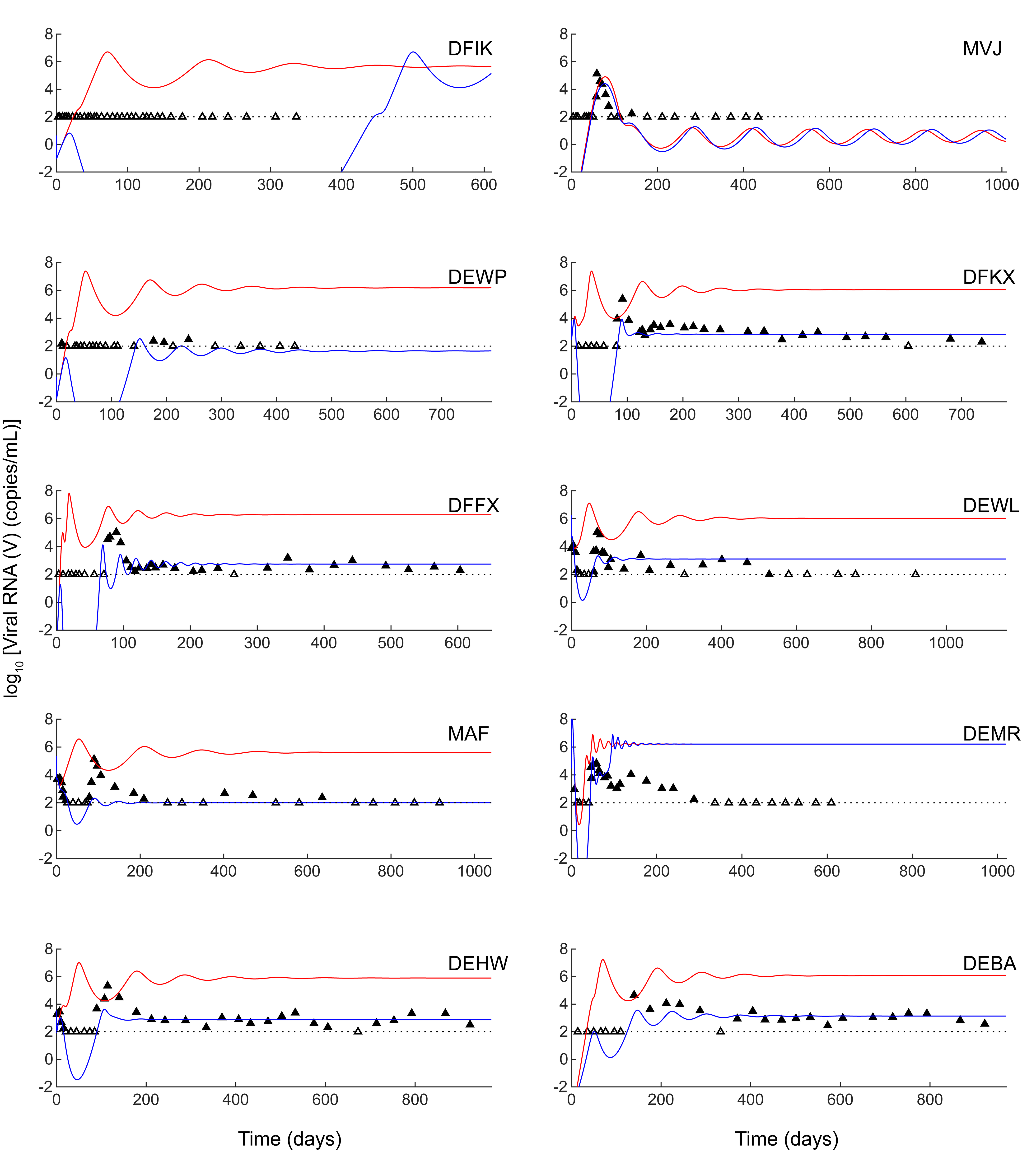

Supplement: S13 Fig — Fitting our model (Eqs 13–20) without enhanced antigen clearance by bNAbs (no AV term in Eq 15) following the procedure outlined in the Methods yielded poor fits (blue) to the data (parameters in S8 Table). Corresponding predictions without treatment are in red. (TIF) [file pcbi.1008064.s013.tif]

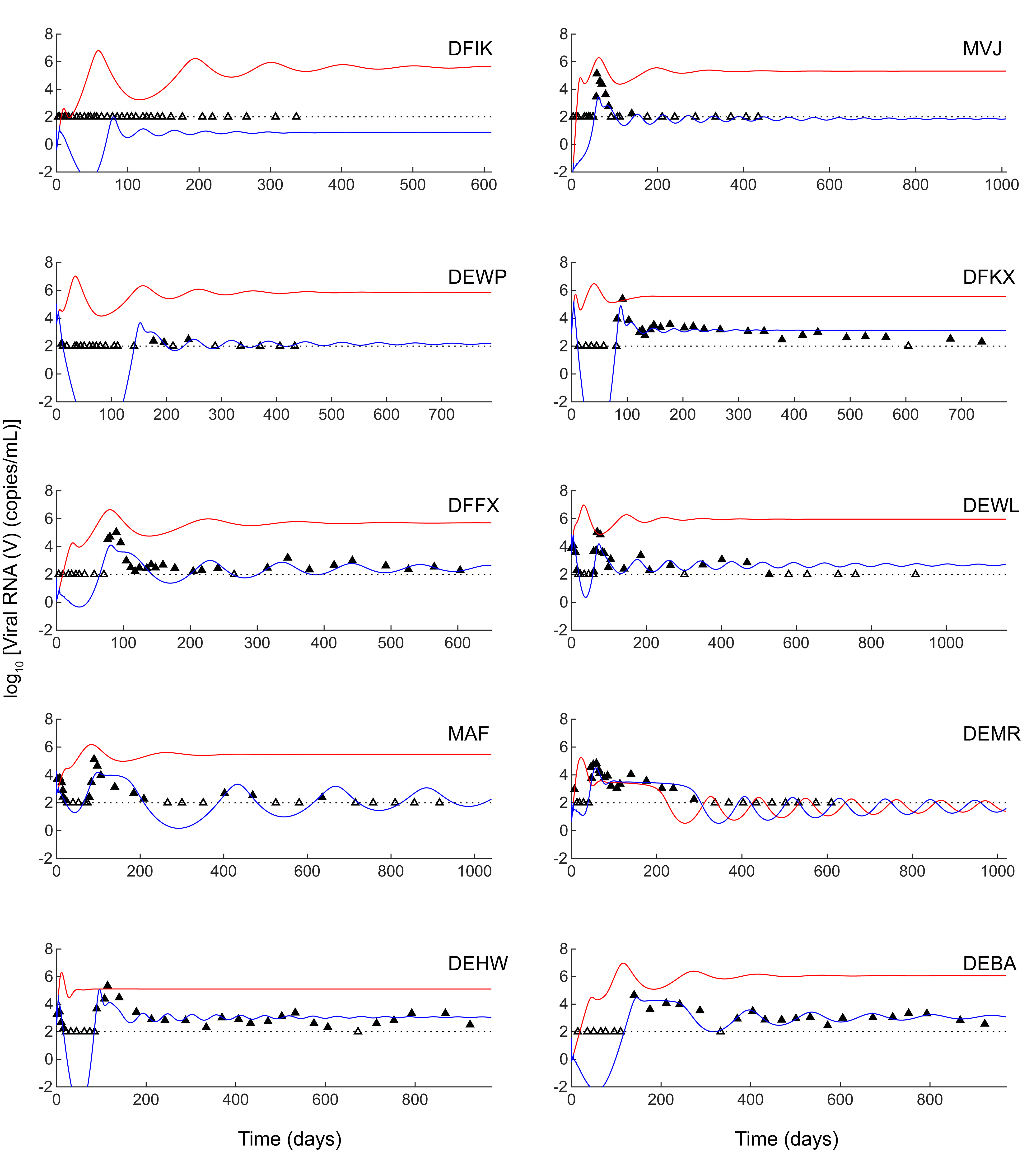

Supplement: S14 Fig — Fitting our model (Eqs 13–20) without enhanced antigen uptake and subsequent effector elicitation by bNAbs (no f*AV term in Eq 16) following the procedure outlined in the Methods yielded poorer fits (blue) to the data (parameters in S9 Table) compared to the main model (Fig 2) and yielded a higher AIC (Table 3). Corresponding model predictions without treatment are in red. (TIF) [file pcbi.1008064.s014.tif]

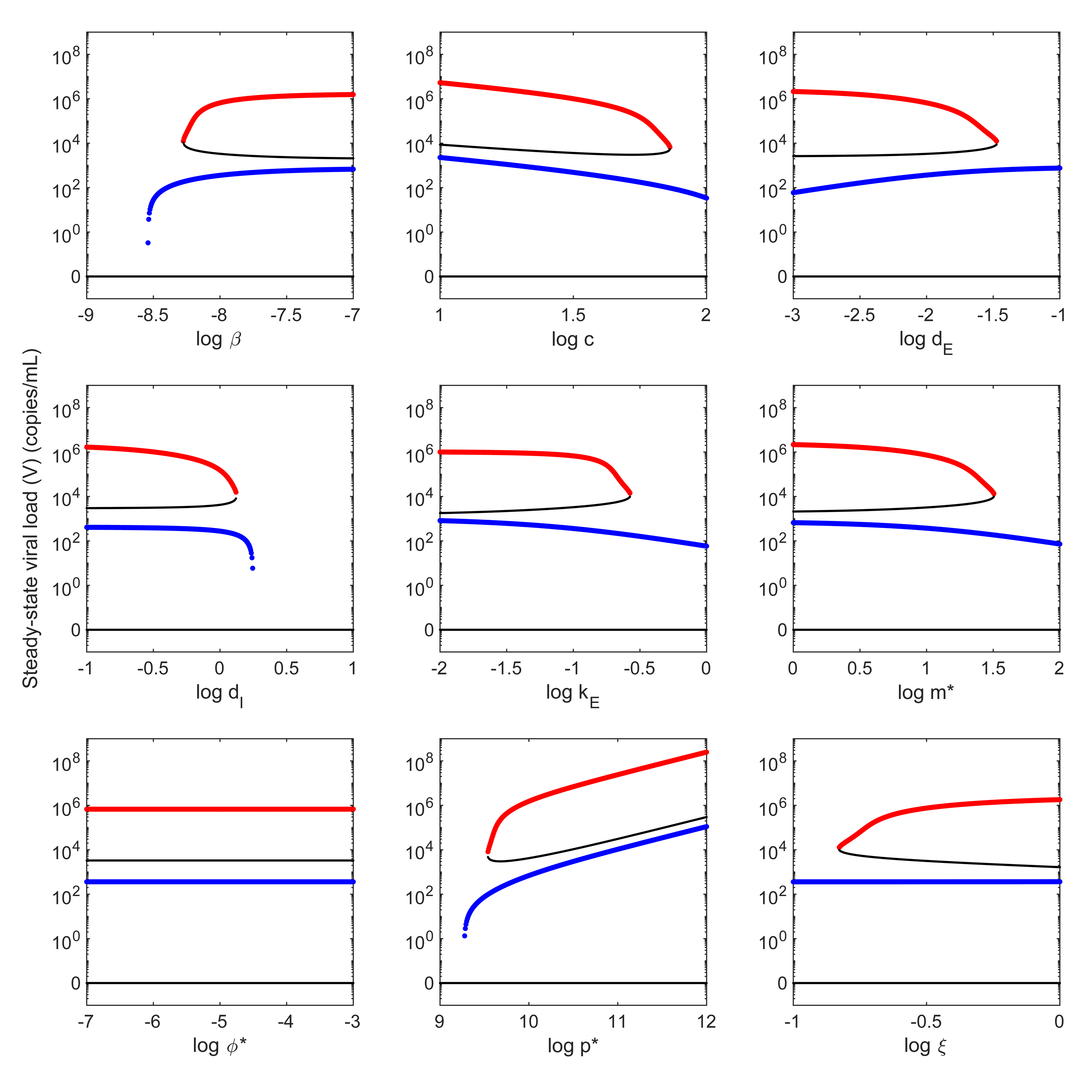

Supplement: S15 Fig — Steady states of our model (Methods) obtained by varying underlying parameters (different panels) one at a time over wide ranges about their values listed in Tables 1 and 2. The stable states of high and low viremia are shown in red and blue, respectively. Black lines represent unstable steady states. Parameters: β—infectivity of virions, c—viral clearance rate, dE—death rate of effectors, dI—death rate of infected CD4+ T cells, kE—proliferation rate of effectors, m*—rate at which effectors kill infected cells, ϕ*—threshold for effector activation as well as level of exhaustion, p*—burst size, and ξ—maximal rate of effector exhaustion. (TIF) [file pcbi.1008064.s015.tif]
