# Supplementary material for: Early exposure to broadly neutralizing antibodies may trigger a dynamical switch from progressive disease to lasting control of SHIV infection
Source: PLoS Comput Biol. 2020 Aug 20;16(8):e1008064. doi: 10.1371/journal.pcbi.1008064 (PMC7462315; doi:10.1371/journal.pcbi.1008064)
Supplement: S1 Table — Parameters pertaining to effector depletion experiments with anti-CD8α and anti-CD8β antibodies were obtained from individual fits (best fits in Fig 2). The units of all the parameters are the same as in Table 2; ζα and ζβ are dimensionless while θm, θα, θβ are in days. (NA—not applicable; see S10 Table). (PDF) [file pcbi.1008064.s016.pdf]

Table S1 Individual parameter estimates for treated macaques obtained by simultaneously fitting our model (Eqs. 13-20) to V, A_1 and A_2 across both untreated macaques and responders (Methods, Figures 2-4). Parameters pertaining to effector depletion experiments with anti-CD8α and anti-CD8β antibodies were obtained from individual fits (best fits in Figure 2). The units of all the parameters are the same as in Table 2; ζ_α and ζ_β are dimensionless while θ_m, θ_α, θ_β are in days. (NA - not applicable)

	DEMR	MVJ	DEWP	DEWL	MAF	DFIK	DFKX	DFFX	DEHW	DEBA
$V(0)$	506.42	0.01	2.35	969.05	665.59	0.12	205.86	0.16	1311.13	11.48
ω_1	2.23	2.59	2.07	2.66	2.27	2.09	1.73	2.10	2.49	2.49
ω_2	1.00	1.09	1.02	1.20	1.06	1.24	1.10	1.22	1.06	0.98
η_1	0.26	0.11	0.04	0.09	0.06	0.13	0.07	0.08	0.06	0.06
η_2	0.23	0.28	0.04	0.09	0.07	0.07	0.12	0.20	0.07	0.05
Vol_1	74.90	138.86	336.77	1187.04	1864.01	52.46	521.95	260.89	433.74	262.41
Vol_2	539.42	695.70	1271.71	347.62	464.55	695.10	1292.10	433.17	972.15	1671.88
k_1	3.66	97.42	80.20	2.63	139.42	58.99	55.13	70.54	91.09	433.80
k_2	694.24	2509.54	46.01	202.07	66.98	5.28	1494.26	1096.20	344.08	41.67
K	59.19	148.67	144.49	308.05	141.89	146.72	32.49	69.74	77.64	35.08
β	1.07×10^{-8}	1.04×10^{-8}	1.08×10^{-8}	1.15×10^{-8}	1.02×10^{-8}	1.05×10^{-8}	1.15×10^{-8}	9.64×10^{-9}	1.01×10^{-8}	9.85×10^{-9}
p^*	4.95×10^{9}	7.15×10^{9}	6.92×10^{9}	6.39×10^{9}	6.61×10^{9}	5.95×10^{9}	5.53×10^{9}	5.76×10^{9}	6.41×10^{9}	7.62×10^{9}
m^*	10.58	10.09	10.48	11.13	12.08	10.90	11.45	10.07	10.34	11.28
d_E	8.36×10^{-3}	6.47×10^{-3}	1.24×10^{-2}	1.48×10^{-2}	5.29×10^{-3}	6.73×10^{-3}	1.22×10^{-2}	1.26×10^{-2}	1.23×10^{-2}	2.03×10^{-2}
ϕ^*	5.02×10^{-5}	2.31×10^{-5}	4.15×10^{-6}	5.45×10^{-5}	4.55×10^{-5}	1.46×10^{-6}	1.18×10^{-4}	4.52×10^{-5}	9.09×10^{-5}	8.84×10^{-5}
ξ	0.17	0.21	0.40	0.22	0.20	0.24	0.21	0.21	0.21	0.25
f^*	3.36×10^{-11}	5.49×10^{-8}	5.08×10^{-9}	7.01×10^{-11}	5.45×10^{-10}	2.91×10^{-9}	1.82×10^{-9}	9.69×10^{-10}	3.07×10^{-10}	1.37×10^{-10}
ζ_α	0.33	0.84	0.99	0.40	0.35	0.90	NA	NA	NA	NA
ζ_β	0.56	0.39	NA	0.46	NA	NA	NA	NA	NA	NA
θ_m	11.86	10.68	18.17	5.33	4.73	3.53	NA	NA	NA	NA
θ_α	607.26	436.54	431.86	918.09	916.21	333.23	NA	NA	NA	NA
θ_β	942.50	939.18	NA	1072.86	NA	NA	NA	NA	NA	NA
