# Supplementary material for: Early exposure to broadly neutralizing antibodies may trigger a dynamical switch from progressive disease to lasting control of SHIV infection
Source: PLoS Comput Biol. 2020 Aug 20;16(8):e1008064. doi: 10.1371/journal.pcbi.1008064 (PMC7462315; doi:10.1371/journal.pcbi.1008064)
Supplement: S2 Table — (PDF) [file pcbi.1008064.s017.pdf]

Table S2 Individual parameter estimates for the ten untreated macaques obtained by simultaneously fitting our model (Eqs. 13-20) to V, A_1 and A_2 across both untreated macaques and responders (Methods and Figure 4).

Macaque	UT1	UT2	UT3	UT4	UT5	UT6	UT7	UT8	UT9	UT10
$V(0)$	52.27	51.93	17.58	3.67	3.22	15.05	1076.07	393.87	243.44	681.12
β	1.06×10^{-8}	1.13×10^{-8}	1.06×10^{-8}	9.61×10^{-9}	9.68×10^{-9}	1.22×10^{-8}	9.74×10^{-9}	1.11×10^{-8}	1.17×10^{-8}	8.86×10^{-9}
p^*	7.83×10^{9}	6.55×10^{9}	7.23×10^{9}	7.85×10^{9}	7.49×10^{9}	5.12×10^{9}	8.56×10^{9}	7.20×10^{9}	7.83×10^{9}	8.07×10^{9}
m^*	11.30	10.66	10.07	10.79	11.83	10.04	10.39	12.06	10.87	11.34
d_E	8.63×10^{-3}	6.93×10^{-3}	7.13×10^{-3}	7.79×10^{-3}	6.57×10^{-3}	8.28×10^{-3}	7.60×10^{-3}	1.23×10^{-2}	4.78×10^{-3}	1.06×10^{-2}
ϕ^*	1.51×10^{-5}	5.44×10^{-6}	1.17×10^{-4}	3.57×10^{-5}	5.85×10^{-5}	1.98×10^{-4}	1.08×10^{-5}	4.49×10^{-5}	2.83×10^{-5}	5.11×10^{-5}
ξ	0.32	0.18	0.54	0.49	0.29	0.43	0.41	0.56	0.33	0.18
