# Supplementary material for: Early exposure to broadly neutralizing antibodies may trigger a dynamical switch from progressive disease to lasting control of SHIV infection
Source: PLoS Comput Biol. 2020 Aug 20;16(8):e1008064. doi: 10.1371/journal.pcbi.1008064 (PMC7462315; doi:10.1371/journal.pcbi.1008064)
Supplement: S3 Table — (PDF) [file pcbi.1008064.s018.pdf]

Table S3 Individual parameter estimates obtained as in Table S1 but with varying effector proliferation rate, k_E (Methods and Figure S8 for details).

	DFIK	MVJ	DEWP	DFKX	DFFX	DEWL	MAF	DEMR	DEHW	DEBA
$V(0)$	2.33×10^{-3}	6.86×10^{-4}	4.45×10^{2}	9.24×10^{6}	4.66×10^{-2}	2.18×10^{4}	1.05×10^{4}	5.29×10^{0}	1.13×10^{3}	4.18×10^{1}
ω_1	1.94	2.26	1.38	2.46	1.48	2.12	1.40	1.72	1.57	1.81
ω_2	1.13	1.57	1.45	1.94	1.43	1.72	1.46	1.27	1.52	1.48
η_1	0.11	0.10	0.04	0.07	0.10	0.08	0.07	0.20	0.07	0.06
η_2	0.07	0.11	0.05	0.20	0.15	0.09	0.08	0.25	0.08	0.06
Vol_1	75.22	151.08	445.13	649.98	209.87	1699.25	1448.49	162.56	404.28	297.63
Vol_2	760.72	1283.45	1112.86	890.94	625.08	338.39	444.12	766.44	876.11	1599.91
k_1	186.99	89.51	263.41	235.32	156.06	190.01	240.82	225.35	214.53	354.58
k_2	157.59	355.60	206.67	192.37	107.03	99.75	158.40	517.34	383.18	315.02
K	46.37	113.75	130.45	59.92	54.12	65.22	56.66	96.71	57.23	25.85
β	2.21×10^{-8}	1.74×10^{-8}	1.79×10^{-8}	1.18×10^{-8}	1.59×10^{-8}	1.26×10^{-8}	1.62×10^{-8}	1.20×10^{-8}	1.41×10^{-8}	1.34×10^{-8}
p^*	5.67×10^{9}	4.15×10^{9}	3.45×10^{9}	5.49×10^{9}	3.90×10^{9}	5.02×10^{9}	4.44×10^{9}	7.15×10^{9}	5.10×10^{9}	5.49×10^{9}
m^*	2.81	3.55	11.30	13.16	9.11	4.67	4.46	3.59	4.29	8.82
d_E	1.46×10^{-3}	8.67×10^{-3}	1.50×10^{-2}	9.75×10^{-3}	1.83×10^{-2}	1.02×10^{-2}	5.14×10^{-3}	1.17×10^{-2}	1.10×10^{-2}	1.36×10^{-2}
k_E	0.34	0.40	0.35	0.44	0.33	0.40	0.37	0.38	0.43	0.32
ϕ^*	1.02×10^{-4}	1.07×10^{-4}	1.24×10^{-4}	3.16×10^{-4}	1.17×10^{-4}	1.34×10^{-4}	2.33×10^{-4}	8.76×10^{-5}	2.75×10^{-4}	1.45×10^{-4}
ξ	1.22	0.49	0.82	1.24	1.13	0.93	0.51	1.24	0.51	0.44
f^*	5.88×10^{-8}	3.18×10^{-9}	6.96×10^{-11}	3.82×10^{-12}	7.21×10^{-10}	8.38×10^{-11}	1.48×10^{-11}	7.71×10^{-9}	1.65×10^{-12}	2.63×10^{-11}
ζ_α	0.57	0.94	0.95	NA	NA	0.85	0.89	0.39	NA	NA
ζ_β	NA	0.76	NA	NA	NA	0.75	NA	0.57	NA	NA
θ_m	2.75	10.87	20.16	NA	NA	14.37	10.75	5.38	NA	NA
θ_α	330.76	432.62	429.95	NA	NA	916.98	915.27	609.98	NA	NA
θ_β	NA	943.54	NA	NA	NA	1077.44	NA	942.11	NA	NA
