# Supplementary material for: Early exposure to broadly neutralizing antibodies may trigger a dynamical switch from progressive disease to lasting control of SHIV infection
Source: PLoS Comput Biol. 2020 Aug 20;16(8):e1008064. doi: 10.1371/journal.pcbi.1008064 (PMC7462315; doi:10.1371/journal.pcbi.1008064)
Supplement: S4 Table — Here, dL was fixed at 0.004 day-1. (PDF) [file pcbi.1008064.s019.pdf]

Table S4 Individual parameter estimates obtained by fitting a basic viral dynamics model without an explicit effector response (Eqs. 25-28; Methods and Figure S9 for details). Here, d_L was fixed at 0.004 day^{-1}.

	DFIK	MVJ	DEWP	DFKX	DFFX	DEWL	MAF	DEMR	DEHW	DEBA
$V(0)$	1.36×10^{-1}	4.75×10^{0}	2.68×10^{7}	3.22×10^{8}	8.40×10^{6}	3.44×10^{7}	5.11×10^{7}	7.17×10^{9}	4.77×10^{7}	7.89×10^{7}
ω_1	2.03	2.23	1.63	2.11	2.06	1.88	1.76	2.54	1.52	2.02
ω_2	1.50	1.12	1.02	1.69	1.36	2.47	1.24	1.49	1.04	1.20
η_1	0.12	0.11	0.04	0.07	0.10	0.09	0.07	0.21	0.07	0.06
η_2	0.08	0.08	0.05	0.24	0.19	0.10	0.06	0.20	0.07	0.08
Vol_1	77.57	119.51	411.73	666.02	199.69	1154.70	1231.05	126.27	402.65	179.70
Vol_2	600.62	1425.75	977.06	534.87	435.31	354.16	535.88	541.10	1119.65	1328.18
k_1	5.18×10^{3}	9.52×10^{-1}	5.97×10^{-3}	5.32×10^{2}	6.42×10^{1}	7.74×10^{2}	6.67×10^{1}	7.47×10^{0}	3.33×10^{4}	6.63×10^{-1}
k_2	9.25×10^{3}	1.15×10^{2}	1.40×10^{5}	1.15×10^{8}	1.71×10^{4}	5.74×10^{5}	2.01E+13	7.38×10^{8}	6.97×10^{4}	3.90×10^{5}
K	8.27×10^{-12}	1.00×10^{-35}	6.06×10^{-52}	3.67×10^{-27}	6.84×10^{-38}	1.68×10^{5}	8.43×10^{12}	4.04×10^{5}	2.17×10^{3}	2.35×10^{2}
β	1.18×10^{-8}	9.84×10^{-9}	1.04×10^{-8}	2.46×10^{-8}	9.56×10^{-9}	6.06×10^{-9}	4.56×10^{-9}	5.39×10^{-9}	5.78×10^{-9}	3.62×10^{-9}
p^*	4.78×10^{9}	3.67×10^{9}	2.08×10^{9}	4.80×10^{9}	5.47×10^{9}	2.94×10^{9}	3.59×10^{9}	2.27×10^{9}	3.26×10^{9}	5.12×10^{9}
f_L	1.29×10^{-1}	6.37×10^{-2}	3.12×10^{-1}	1.24×10^{-1}	3.09×10^{-1}	5.33×10^{-2}	3.98×10^{-2}	6.18×10^{-2}	2.20×10^{-1}	1.04×10^{-1}
ψ	2.87×10^{-4}	2.39×10^{-4}	2.32×10^{-4}	2.85×10^{-4}	3.35×10^{-4}	2.95×10^{-4}	6.02×10^{-5}	1.83×10^{-3}	3.61×10^{-4}	8.09×10^{-5}
a	1.48×10^{-2}	4.68×10^{-3}	9.90×10^{-3}	3.02×10^{-3}	3.69×10^{-3}	1.21×10^{-2}	3.35×10^{-3}	3.34×10^{-2}	9.75×10^{-3}	3.89×10^{-3}
