# Supplementary material for: Early exposure to broadly neutralizing antibodies may trigger a dynamical switch from progressive disease to lasting control of SHIV infection
Source: PLoS Comput Biol. 2020 Aug 20;16(8):e1008064. doi: 10.1371/journal.pcbi.1008064 (PMC7462315; doi:10.1371/journal.pcbi.1008064)
Supplement: S6 Table — (PDF) [file pcbi.1008064.s021.pdf]

Table S6 Individual parameter estimates as obtained in Table S1 but with the Hill coefficient $n = 3$ (Methods and Figure S11 for details).

	DFIK	MVJ	DEWP	DFKX	DFFX	DEWL	MAF	DEMR	DEHW	DEBA
$V(0)$	6.34×10^{0}	1.33×10^{-1}	3.53×10^{2}	9.87×10^{2}	1.00×10^{3}	9.28×10^{2}	9.99×10^{2}	9.99×10^{2}	1.00×10^{3}	9.53×10^{2}
ω_1	1.78	1.27	1.58	1.32	1.89	1.30	1.80	1.85	1.59	1.72
ω_2	1.78	1.81	1.77	1.88	1.96	1.75	2.31	1.90	2.44	1.97
η_1	0.12	0.13	0.04	0.07	0.10	0.08	0.06	0.21	0.07	0.06
η_2	0.08	0.15	0.05	0.18	0.17	0.09	0.07	0.14	0.07	0.07
Vol_1	80.20	80.30	548.87	608.11	167.20	1630.38	2194.97	117.92	387.75	243.09
Vol_2	601.75	1470.23	1037.82	866.97	564.90	369.15	477.42	832.63	1222.64	1501.78
k_1	54.91	33.41	21.63	75.68	24.69	47.39	46.89	59.66	50.04	58.85
k_2	87.54	377.98	162.26	177.43	1349.73	377.25	64.73	512.79	303.04	505.15
K	3067.59	104.77	570.35	158.55	488.02	1468.32	28.57	453.75	141.67	56.91
β	6.53×10^{-9}	1.13×10^{-8}	4.68×10^{-9}	1.11×10^{-8}	6.45×10^{-9}	1.47×10^{-8}	5.38×10^{-9}	1.44×10^{-8}	1.47×10^{-8}	8.28×10^{-9}
p^*	5.20×10^{9}	4.15×10^{9}	6.82×10^{9}	4.52×10^{9}	5.79×10^{9}	4.45×10^{9}	8.52×10^{9}	8.91×10^{9}	4.32×10^{9}	5.61×10^{9}
m^*	16.26	15.01	28.81	17.39	25.29	17.42	13.23	36.92	13.29	29.18
d_E	1.06×10^{-3}	1.97×10^{-3}	2.74×10^{-3}	8.47×10^{-3}	7.99×10^{-3}	5.30×10^{-3}	1.44×10^{-3}	1.64×10^{-2}	9.84×10^{-3}	8.79×10^{-3}
ϕ^*	2.65×10^{-5}	5.78×10^{-5}	7.59×10^{-5}	1.96×10^{-4}	1.97×10^{-4}	7.21×10^{-5}	1.05×10^{-4}	5.06×10^{-5}	2.58×10^{-4}	3.29×10^{-4}
ξ	1.08	0.21	3.92	1.28	1.64	0.26	0.26	0.52	0.24	1.51
f^*	5.28×10^{-7}	3.22×10^{-8}	3.67×10^{-8}	6.85×10^{-9}	5.92×10^{-9}	1.17×10^{-9}	3.69×10^{-9}	3.29×10^{-10}	9.08×10^{-10}	9.36×10^{-9}
ζ_α	0.99	0.00	0.38	NA	NA	1.00	0.00	1.00	NA	NA
ζ_β	NA	0.67	NA	NA	NA	0.44	NA	0.34	NA	NA
θ_m	30.00	23.16	25.06	NA	NA	10.74	8.36	13.69	NA	NA
θ_α	326.00	425.05	424.00	NA	NA	916.51	913.15	613.24	NA	NA
θ_β	NA	936.50	NA	NA	NA	1074.76	NA	947.50	NA	NA
