# Supplementary material for: Early exposure to broadly neutralizing antibodies may trigger a dynamical switch from progressive disease to lasting control of SHIV infection
Source: PLoS Comput Biol. 2020 Aug 20;16(8):e1008064. doi: 10.1371/journal.pcbi.1008064 (PMC7462315; doi:10.1371/journal.pcbi.1008064)
Supplement: S7 Table — (PDF) [file pcbi.1008064.s022.pdf]

Table S7 Individual parameter estimates as obtained in Table S1 but with the Hill coefficient $n = 1$ (Methods and Figure S12 for details).

	DFIK	MVJ	DEWP	DFKX	DFFX	DEWL	MAF	DEMR	DEHW	DEBA
$V(0)$	7.84×10^{-3}	9.42×10^{-3}	1.58×10^{3}	2.67×10^{-1}	1.42×10^{-1}	4.67×10^{4}	1.68×10^{5}	7.01×10^{2}	3.99×10^{3}	3.00×10^{0}
ω_1	1.31	2.21	0.97	2.00	1.54	1.43	0.85	1.61	2.27	1.61
ω_2	0.89	1.67	1.04	2.43	2.32	1.58	1.08	2.05	1.48	1.43
η_1	0.12	0.10	0.04	0.06	0.10	0.07	0.07	0.28	0.07	0.06
η_2	0.07	0.25	0.05	0.25	0.14	0.09	0.07	0.27	0.07	0.06
Vol_1	64.72	138.60	502.26	627.41	136.64	1783.29	1487.30	47.12	400.49	247.16
Vol_2	641.78	834.52	1088.40	683.72	637.74	317.29	555.65	479.13	1036.00	1444.39
k_1	0.81	0.02	0.35	0.70	0.28	2.92	0.30	0.29	0.47	0.13
k_2	56.94	3027.28	4.21	138.79	55.87	39.10	50.87	65.15	41766.23	1047.63
K	1.85×10^{-5}	6.07×10^{-8}	2.29×10^{-3}	7.54×10^{-4}	0.04	162.74	153.32	35.28	2364.55	36.66
β	8.72×10^{-9}	8.46×10^{-9}	7.23×10^{-9}	9.60×10^{-9}	6.89×10^{-9}	1.80×10^{-8}	7.61×10^{-9}	5.39×10^{-9}	3.10×10^{-8}	6.00×10^{-9}
p^*	4.65×10^{9}	6.94×10^{9}	3.13×10^{9}	3.04×10^{9}	4.26×10^{9}	3.44×10^{9}	5.88×10^{9}	5.20×10^{9}	2.74×10^{9}	8.55×10^{9}
m^*	2.73	41.58	1.81	2.71	6.78	483.58	5180.91	26.52	9.59	0.30
d_E	3.82×10^{-2}	7.74×10^{-4}	2.33×10^{-2}	4.21×10^{-1}	3.51×10^{-3}	8.25×10^{-4}	2.81×10^{-4}	1.63×10^{-5}	2.99×10^{-5}	6.49×10^{-4}
ϕ^*	3.11×10^{-3}	1.62×10^{-3}	3.06×10^{-3}	1.44×10^{-4}	6.59×10^{-5}	5.81×10^{-4}	6.29×10^{-3}	3.72×10^{-3}	1.01×10^{-4}	2.34×10^{-5}
ξ	0.65	0.32	0.02	0.17	0.09	0.19	0.80	0.04	0.06	0.07
f^*	1.93×10^{-4}	2.12×10^{-5}	1.38×10^{-5}	2.67×10^{-9}	4.15×10^{-9}	3.50×10^{-13}	1.24×10^{-10}	8.23×10^{-6}	1.92×10^{-11}	7.18×10^{-8}
