# Supplementary material for: Early exposure to broadly neutralizing antibodies may trigger a dynamical switch from progressive disease to lasting control of SHIV infection
Source: PLoS Comput Biol. 2020 Aug 20;16(8):e1008064. doi: 10.1371/journal.pcbi.1008064 (PMC7462315; doi:10.1371/journal.pcbi.1008064)
Supplement: S8 Table — (PDF) [file pcbi.1008064.s023.pdf]

Table S8 Individual parameter estimates for treated macaques obtained by simultaneously fitting models without enhanced antigen clearance by bNAbs (no AV term in Eq. 15) to V, A_1 and A_2 across both untreated macaques and responders (Methods and Figure S13 for details).

	DFIK	MVJ	DEWP	DFKX	DFFX	DEWL	MAF	DEMR	DEHW	DEBA
$V(0)$	4.42×10^{0}	4.52×10^{-5}	6.20×10^{-1}	6.81×10^{3}	4.39×10^{-3}	1.72×10^{6}	2.35×10^{5}	8.76×10^{4}	3.48×10^{3}	1.53×10^{-3}
ω_1	2.33	1.71	2.54	2.16	2.02	2.57	2.09	1.51	2.33	1.78
ω_2	1.46	1.26	1.17	1.05	1.09	1.17	1.35	0.99	1.12	1.20
η_1	0.11	0.12	0.04	0.07	0.11	0.07	0.07	0.24	0.07	0.06
η_2	0.07	0.26	0.04	0.23	0.23	0.09	0.08	0.18	0.07	0.06
Vol_1	90.40	90.56	458.29	686.27	128.48	2668.51	1590.33	76.79	433.49	252.96
Vol_2	854.29	818.14	1006.27	736.39	265.97	372.44	441.28	529.42	1011.31	1349.65
k_1	5.27×10^{-10}	6.41E-18	2.91×10^{-2}	5.29×10^{-5}	2.94×10^{-5}	5.58×10^{-12}	3.90×10^{-5}	1.48×10^{-9}	8.12×10^{-9}	1.02×10^{1}
k_2	68.47	34.20	88.56	13.95	106.02	30.71	34.26	13.37	57.79	54.87
K	36852.02	382.85	5047.78	546.43	984.93	22845.91	58520.72	1116.77	5159.19	517.12
β	1.29×10^{-8}	1.69×10^{-8}	6.28×10^{-9}	1.37×10^{-8}	1.44×10^{-8}	6.06×10^{-9}	9.95×10^{-9}	3.28×10^{-8}	8.94×10^{-9}	7.55×10^{-9}
p^*	2.39×10^{9}	2.08×10^{9}	6.23×10^{9}	3.99×10^{9}	8.34×10^{9}	5.64×10^{9}	2.98×10^{9}	1.49×10^{10}	3.89×10^{9}	4.82×10^{9}
m^*	13.17	1.56	12.47	18.64	34.81	8.61	5.18	310.52	6.22	12.38
d_E	5.29×10^{-3}	6.33×10^{-3}	1.61×10^{-2}	3.78×10^{-2}	3.50×10^{-2}	5.31×10^{-2}	4.81×10^{-2}	8.96×10^{-4}	3.05×10^{-2}	1.66×10^{-2}
ϕ^*	6.41×10^{-6}	1.52×10^{-6}	7.74×10^{-6}	1.41×10^{-4}	3.65×10^{-5}	1.91×10^{-4}	2.50×10^{-5}	5.36×10^{-5}	1.28×10^{-4}	3.38×10^{-4}
ξ	6.14	0.10	7.91	10.32	3.80	9.74	4.92	0.30	5.46	28.05
f^*	5.88×10^{-6}	1.42×10^{-6}	2.95×10^{-7}	2.37×10^{-7}	8.33×10^{-6}	8.14×10^{-7}	1.24×10^{-6}	7.19×10^{-7}	2.22×10^{-7}	3.35×10^{-7}
