# Supplementary material for: Early exposure to broadly neutralizing antibodies may trigger a dynamical switch from progressive disease to lasting control of SHIV infection
Source: PLoS Comput Biol. 2020 Aug 20;16(8):e1008064. doi: 10.1371/journal.pcbi.1008064 (PMC7462315; doi:10.1371/journal.pcbi.1008064)
Supplement: S9 Table — (PDF) [file pcbi.1008064.s024.pdf]

Table S9 Individual parameter estimates for treated macaques obtained by simultaneously fitting models without enhanced antigen uptake and subsequent effector elicitation by bNAbs (no $f^{*}AV$ term in Eq. 16) to V, A_1 and A_2 across both untreated macaques and responders (Methods and Figure S14 for details).

	DFIK	MVJ	DEWP	DFKX	DFFX	DEWL	MAF	DEMR	DEHW	DEBA
$V(0)$	1.07×10^{1}	5.51×10^{-3}	1.12×10^{5}	1.29×10^{4}	5.09×10^{1}	8.05×10^{4}	1.99×10^{4}	1.36×10^{2}	6.62×10^{3}	3.30×10^{1}
ω_1	1.57	1.53	1.18	1.43	1.54	1.51	1.46	1.36	1.30	1.40
ω_2	2.25	1.79	1.48	1.75	2.11	1.95	2.04	1.29	2.00	1.86
η_1	0.12	0.11	0.04	0.08	0.10	0.08	0.08	0.22	0.07	0.06
η_2	0.07	0.11	0.05	0.19	0.19	0.10	0.08	0.17	0.08	0.05
Vol_1	62.50	114.80	445.53	484.85	192.72	2002.30	1616.27	111.04	307.54	237.20
Vol_2	734.61	1366.46	903.80	911.61	477.84	295.37	467.38	773.07	893.40	1902.33
k_1	64.61	49.97	49.66	67.34	47.38	54.47	39.34	55.81	93.15	37.50
k_2	190.11	401.45	88.00	122.42	114.03	96.48	51.62	160.31	97.58	208.22
K	97.45	47.13	28.06	27.34	48.03	237.89	84.13	54.24	70.78	124.71
β	1.01×10^{-8}	8.88×10^{-9}	6.78×10^{-9}	7.37×10^{-9}	6.01×10^{-9}	4.61×10^{-9}	6.80×10^{-9}	6.76×10^{-9}	7.69×10^{-9}	3.05×10^{-9}
p^{*}	5.41×10^{9}	7.30×10^{9}	7.20×10^{9}	1.04×10^{10}	6.21×10^{9}	1.08×10^{10}	4.27×10^{9}	6.97×10^{9}	8.75×10^{9}	9.68×10^{9}
m^{*}	9.92	14.93	11.91	12.83	11.64	11.44	7.33	8.40	11.58	7.97
d_E	2.48×10^{-2}	1.06×10^{-2}	1.23×10^{-2}	2.20×10^{-2}	8.60×10^{-3}	7.85×10^{-3}	4.23×10^{-3}	5.72×10^{-3}	1.20×10^{-2}	8.58×10^{-3}
ϕ^{*}	6.86×10^{-7}	7.79×10^{-6}	1.71×10^{-5}	5.85×10^{-5}	6.23×10^{-5}	4.38×10^{-5}	1.51×10^{-4}	9.75×10^{-6}	8.08×10^{-5}	1.84×10^{-4}
ξ	0.58	0.27	0.52	0.34	0.51	0.32	0.33	0.14	0.20	1.04
