# Supplementary material for: Early exposure to broadly neutralizing antibodies may trigger a dynamical switch from progressive disease to lasting control of SHIV infection
Source: PLoS Comput Biol. 2020 Aug 20;16(8):e1008064. doi: 10.1371/journal.pcbi.1008064 (PMC7462315; doi:10.1371/journal.pcbi.1008064)
Supplement: S10 Table — Macaques to which only anti-CD8α Abs, or both anti-CD8α and anti-CD8β Abs were administered, have their IDs colored blue and purple, respectively. Non-controller macaque IDs are colored orange and their data was not considered for fitting. (PDF) [file pcbi.1008064.s025.pdf]

Table S10 Summary table of macaques from Nishimura *et al.* which were subjected to early bNAb therapy, with Macaque ID, initial viral inoculum, route of administration, whether anti-CD8α Ab- and anti-CD8β Ab-mediated effector depletion was performed, and whether these macaques regained viremic control subsequently. Macaques to which only anti-CD8α Abs, or both anti-CD8α and anti-CD8β Abs were administered, have their IDs colored blue and purple, respectively. Non-controller macaque IDs are colored orange and their data was not considered for fitting.

Macaque ID	Viral inoculum ($TCID_{50}$)	Route of administration	Controller	Anti-CD8α Abs	Anti-CD8β Abs	Regained control
DEMR	100	Intravenous	Yes	Yes	Yes	Yes
MVJ	1000	Intrarectal	Yes	Yes	Yes	Yes
DEWP	1000	Intrarectal	Yes	Yes	–	Yes
DEWL	1000	Intravenous	Yes	Yes	Yes	Yes
MAF	1000	Intravenous	Yes	Yes	–	Yes
DFIK	1000	Intrarectal	Yes	Yes	–	No
DFKX	1000	Intrarectal	Yes	–	–	–
DFFX	1000	Intrarectal	Yes	–	–	–
DEHW	100	Intravenous	Yes	–	–	–
DEBA	100	Intravenous	Yes	–	–	–
DEPH	1000	Intrarectal	No	–	–	–
DF06	1000	Intravenous	No (AIDS)	–	–	–
DELV	1000	Intravenous	No	–	–	–
