# Supplementary material for: Early exposure to broadly neutralizing antibodies may trigger a dynamical switch from progressive disease to lasting control of SHIV infection
Source: PLoS Comput Biol. 2020 Aug 20;16(8):e1008064. doi: 10.1371/journal.pcbi.1008064 (PMC7462315; doi:10.1371/journal.pcbi.1008064)
Supplement: S5 Table — (PDF) [file pcbi.1008064.s020.pdf]

Table S5 Individual parameter estimates obtained by fitting the post-ART control model (Eqs. 31-35; Methods and Figure S10 for details).

	DFIK	MVJ	DEWP	DFKX	DFFX	DEWL	MAF	DEMR	DEHW	DEBA
$V(0)$	9.86×10^{2}	1.98×10^{3}	1.66×10^{2}	2.94×10^{2}	1.53×10^{-2}	6.01×10^{4}	3.57×10^{3}	2.47×10^{3}	1.61×10^{4}	1.42×10^{3}
ω_1	1.71	2.09	1.10	1.51	1.60	2.55	1.36	1.58	1.65	1.72
ω_2	1.14	1.32	1.12	1.48	0.85	0.86	0.84	1.17	1.74	1.19
η_1	0.12	0.10	0.04	0.07	0.10	0.08	0.08	0.26	0.07	0.06
η_2	0.07	0.23	0.04	0.25	0.16	0.10	0.07	0.18	0.07	0.07
Vol_1	58.22	156.47	465.69	618.50	220.32	1644.68	1201.31	53.05	323.90	276.45
Vol_2	764.22	901.37	1250.82	575.51	481.40	258.48	418.49	782.81	1066.82	1272.74
k_1	0.00	0.00	2510.94	0.00	0.00	0.00	30708.34	0.00	0.00	0.00
k_2	1588.14	294.24	162.13	1792.36	383.87	232.45	111.35	181.36	392.91	291.91
K	457.27	798.00	139.83	250.77	2241.92	1516.07	1354.24	1359.91	133.12	81.76
β	1.04×10^{-8}	1.94×10^{-8}	5.53×10^{-8}	1.90×10^{-8}	1.87×10^{-8}	2.00×10^{-8}	4.85×10^{-8}	1.23×10^{-8}	2.03×10^{-8}	1.22×10^{-8}
p^*	2.53×10^{9}	1.87×10^{9}	2.35×10^{9}	2.12×10^{9}	2.17×10^{9}	1.48×10^{9}	2.19×10^{9}	2.27×10^{9}	1.55×10^{9}	2.75×10^{9}
m^*	0.15	12.71	0.56	0.23	2.37	0.06	0.22	0.28	1.25	0.21
d_E	1.35×10^{-2}	2.44×10^{-3}	1.07×10^{-3}	9.04×10^{-2}	1.12×10^{-2}	7.22×10^{-2}	2.01×10^{-2}	2.02×10^{-2}	4.16×10^{-5}	4.25×10^{-2}
ϕ_1^*	2.27×10^{-1}	5.27×10^{-3}	5.53×10^{-7}	2.05×10^{-5}	4.21×10^{-6}	5.32×10^{-6}	9.18×10^{-6}	9.94×10^{-5}	7.58×10^{-6}	3.79×10^{-5}
ξ	0.07	0.07	0.07	0.07	0.12	0.10	0.11	0.08	0.07	0.10
ϕ_2^*	7.48×10^{-6}	5.30×10^{-7}	3.95×10^{-6}	1.55×10^{-3}	4.62×10^{-11}	3.44×10^{-4}	1.27×10^{-4}	6.07×10^{-5}	8.31×10^{-7}	1.08×10^{-4}
f^*	0	0	0	0	0	0	0	0	0	0
